# Activation of pentafluoropropane isomers at a nanoscopic aluminum chlorofluoride: hydrodefluorination versus dehydrofluorination

**DOI:** 10.3762/bjoc.16.213

**Published:** 2020-10-23

**Authors:** Maëva-Charlotte Kervarec, Thomas Braun, Mike Ahrens, Erhard Kemnitz

**Affiliations:** 1 Department of Chemistry, Humboldt-Universität zu Berlin, Brook-Taylor-Straße 2, 12489 Berlin, Germany

**Keywords:** aluminum fluoride, C–F bond activation, dehydrofluorination, hydrodefluorination, hydrofluorocarbons

## Abstract

The hydrofluorocarbon 245 isomers, 1,1,1,3,3-pentafluoropropane, 1,1,1,2,2- pentafluoropropane, and 1,1,1,2,3-pentafluoropropane (HFC-245fa, HFC-245cb, and HFC-245eb) were activated through C–F bond activations using aluminium chlorofluoride (ACF) as a catalyst. The addition of the hydrogen source Et_3_SiH is necessary for the activation of the secondary and tertiary C–F bonds. Multiple C–F bond activations such as hydrodefluorinations and dehydrofluorinations were observed, followed by hydroarylation and Friedel–Crafts-type reactions under mild conditions.

## Introduction

Hydrofluorocarbons (HFCs) have been intensively used in daily life, mainly due to their excellent properties in refrigeration applications [1–3]. In the past, HFCs were considered as replacements that do not deplete ozone for chlorofluorocarbons (CFCs) and hydrochlorofluorocarbons (HCFCs), which have been strictly regulated by the Montreal protocol [4–6]. However, due to the high global warming potential (GWP), HFCs have also been included in the Montreal protocol in 2019 (Kigali amendment) and have to be phased out [7–10].

On the other hand, HFCs are valuable starting compounds or intermediate products for the synthesis of hydrofluoroolefins (HFOs), which have been regarded as the next generation of refrigerants, exhibiting zero ozone depletion potential (ODP) and a negligible GWP [11–13]. A considerable amount of studies has been carried out to synthesize HFOs under mild conditions [11,14–16]. Among them are routes to access 2,3,3,3-tetrafluoropropene and 1,3,3,3-tetrafluoropropene (HFO-1234yf and HFO-1234ze), for which numerous patents suggest synthetic pathways and showcase the reactivity [12–13,15]. One possibility for the preparation includes the conversion of pentafluoropropanes (HFC-245 isomers) using chromia-based catalysts, or metal chloride/fluoride (AlF_3_, MgF_2_)-supported catalysts at elevated temperatures (350 °C) [11,14–15,17–18]. The group of Lu recently reported the gas-phase transformation of 1,1,1,3,3-pentafluoropropane (HFC-245eb) into 1,3,3,3-tetrafluoropropene (HFO-1234ze) using mesoporous nanoscopic aluminum fluoride-based catalysts [19]. The catalysts were prepared via a sol–gel process in the presence of polyols, allowing for the evolution of a large surface area and improved acidic properties when compared to fluorinated Cr_2_O_3_ or traditional β-AlF_3_ catalysts. At a reaction temperature set at 280 °C, the conversion of 1,1,1,2,2- pentafluoropropane (HFC-245fa) into the 1,3,3,3-tetrafluoropropene (HFO-1234ze) varied between 50 and 60%, depending on the conditions used to synthesize the catalyst, reaching almost full selectivity. The harsh conditions are in part needed due to the high dissociation energy of C–F bonds, and in general, C–F activation steps are considered to be challenging [20–27].

**Scheme 1:**
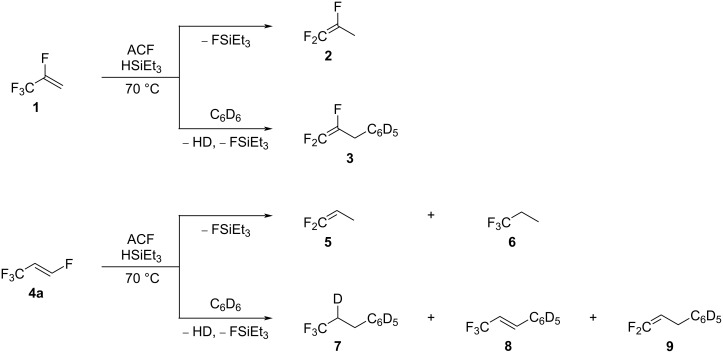
Reactivity of tetrafluoropropanes HFO-1234yf (**1**) (top) and HFO-1234ze (**4a**) (bottom) in the presence of ACF as the catalyst and HSiEt_3_ as a hydrogen source [16].

Solid Lewis acids with a high fluoride ion affinity as catalysts are useful tools for C–F bond activation reactions since the Lewis acidic centers can induce dehydrofluorination reactions, involving the abstraction of a fluoride ion by heterolytic bond cleavage [28–31]. AlF_3_-based catalysts are among the strongest Lewis acidic materials. They exhibit an effective activity in C–F bond conversion reactions and are widely investigated [16,28,32–39]. Especially microporous aluminum chlorofluoride (ACF, AlCl_x_F_3−_*_x_*; *x* = 0.05–0.3), which has a large surface area (>200 m^2^g^−1^) and was patented by Dupont in 1992, has been extensively studied [40–46]. It is an amorphous aluminum fluoride doped with chlorine atoms which causes a distortion of the structure resulting in the amorphicity and high Lewis acidity of the compound. The reactivity of ACF towards C–F bond activations was deeply investigated. For instance, the activation of fluoromethanes was observed at ACF in the presence of HSiEt_3_ as a hydrogen source to produce, in the presence of benzene as the solvent, Friedel–Crafts products as main compounds [47]. In contrast, the hydrodefluorination products were generated in the absence of benzene. Thermodynamically, the generation of strong H–F, Al–F, or Si–F bonds can enforce an activation of C–F bonds under mild conditions, and hence the addition of the silane HSiEt_3_ as a hydrogen source [27,48–49]. More recently, ACF was shown to efficiently convert the fluoroalkenes HFO-1234yf (**1**) and HFO-1234ze (**4a**) in the presence of the hydrogen source HSiEt_3_ into the hydrodefluorination or Friedel–Crafts products (Scheme 1) [16].

The activation of fluoropentane was achieved using a modified ACF, loaded with germane or silane [39]. When silane was immobilized at the surface of ACF in the presence of benzene, Friedel–Crafts products were again generated. In comparison, when ACF was loaded with germane, dehydrofluorination products were detected. Besides, 2-chloro-1,1,1,2-tetrafluoropropane (HCFC-244bb) was as well effectively activated at ACF to yield the corresponding dehydrofluorination product 2-chloro-3,3,3-trifluoropropene (HFO-1233xf) without the use of any additional hydrogen source [50]. In the presence of silane and ACF, HFO-1233xf was further activated via allylic hydrodefluorination.

In this paper, we report on the reactivity of ACF towards hydrofluorocarbons, and in particular, the pentafluoropropane isomers (HFC-245). Effective hydrodefluorination and dehydrofluorination steps of pentafluoropropane isomers in the presence of Et_3_SiH as a hydrogen source at mild conditions are described.

## Results and Discussion

### Activation of 1,1,1,2,3-pentafluoropropane (HFC-245eb, **10a**)

**Scheme 2:**
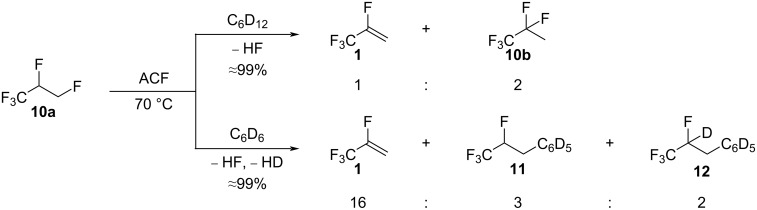
Reactivity of **10a** in the presence of ACF as the catalyst in C_6_D_12_ (top) or C_6_D_6_ (bottom) as solvents.

The treatment of 1,1,1,2,3-pentafluoropropane (**10a**) with ACF at 70 °C in C_6_D_12_ gave the dehydrofluorination product 2,3,3,3-tetrafluoropropene (HFO-1234yf, **1**) and the isomerization product 1,1,1,2,2-pentafluoropropane (HFC-245cb, **10b**) in a 1:2 ratio (Scheme 2, top) with almost full conversion. The group of Kemnitz previously showed that **1** and **10b** can be in an equilibrium when HF is present in the reaction mixture [33]. It was demonstrated that starting from 2-chloro-3,3,3-trifluoropropene (HFO-1233xf) in the presence of fluorinated Cr_2_O_3_ as a catalyst and HF, 2,3,3,3-tetrafluoropropene (HFO-1234yf, **1**) is generated by the replacement of the chlorine substituent with a fluorine atom, and is further transformed by HF addition into 1,1,1,2,2-pentafluoropropane (HFC-245cb, **10b**) [33].

When the aromatic solvent C_6_D_6_ was used instead of C_6_D_12_, **10a** was once more transformed into **1** as the main compound, together with traces of the Friedel–Crafts product CF_3_CFHCH_2_C_6_D_5_ (**11**) and the hydroarylation product CF_3_CFDCH_2_C_6_D_5_ (**12**) (Scheme 2, bottom), with only 22% conversion. The low conversion in C_6_D_6_ could be a consequence of a possible interaction of the aromatic solvent with the surface of ACF, which would result in the blocking of the acidic sites, and thus hamper the adsorption of the substrates. Indeed, in a previous study, a pulse TA experiment suggested the presence of a strong interaction between benzene and the surface of ACF [38]. This result was further confirmed by ^1^H MAS NMR spectroscopy.

Note, that **10a** was activated under mild conditions without the use of an additional hydrogen source, which often has been added for the activation of C–F bonds at ACF [16,39,47]. Several patents cover the transformation of **10a** by dehydrofluorination at chromia-based catalysts, but the reaction temperatures were above 200 °C [17,51–52].

**Scheme 3:**
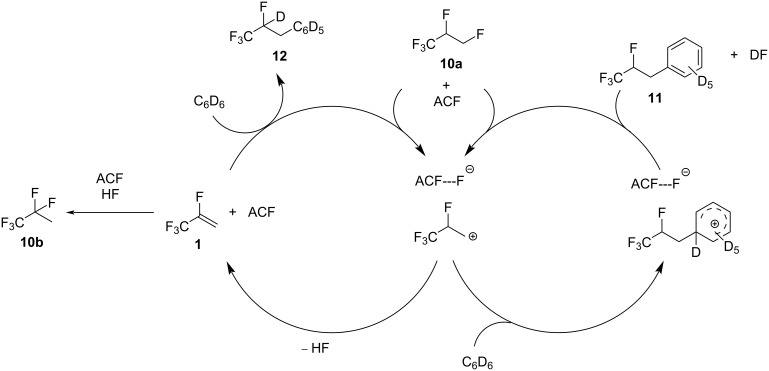
Proposed catalytic cycle of the transformation of **10a** in C_6_D_12_ and C_6_D_6_ in the presence of ACF as the catalyst.

Mechanistically, an abstraction of a fluorine from the CH_2_F group by the surface of ACF can occur, generating carbenium-like species and surface fluorides (Scheme 3). Via HF elimination, the olefin **1** can be produced, followed by a refluorination of the double bond by the released HF, generating **10b** (Scheme 3, left). In the presence of C_6_D_6_, the hydroarylation product **12** can be generated from **1** at the ACF surface. Alternatively, the aromatic solvent can also attack the carbenium-like species, producing a zwitterionic Wheeland intermediate, which can release the Friedel–Crafts product **11** and DF to regenerate the catalyst (Scheme 3, right).

Although a hydrogen source was not needed to accomplish the activation of **10a**, it was of interest to introduce a silane, because as mentioned above, recent reports showed that the activation of various substrates treated with ACF was indeed promoted by the presence of silanes [16,39,47]. Thus, the experiments were also conducted in the presence of HSiEt_3_, either in a solvent (C_6_D_6_ or C_6_D_12_), or in neat silane under similar conditions (all reactions were carried out at 70 °C and monitored for 7 days).

**Scheme 4:**
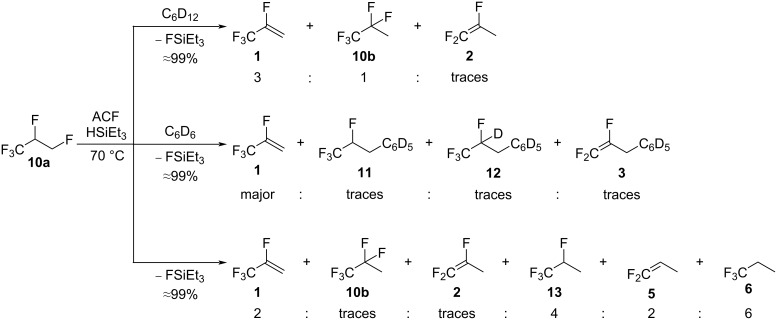
Reactivity of **10a** in the presence of ACF as the catalyst and HSiEt_3_ as a hydrogen source in C_6_D_12_ (top) or C_6_D_6_ (middle) as solvents or in neat silane (bottom).

The treatment of **10a** and HSiEt_3_ in C_6_D_12_ generated **1** and **10b** again, in addition to traces of 1,1,2-trifluoropropene (**2**, Scheme 4, top). The ratio between the olefin **1** and the refluorination product **10b** observed in the presence of HSiEt_3_ was 3:1, whereas, without the silane, a ratio of 1:2 was detected. This difference in the ratio might relate to the amount of HF present in the reaction mixture, which would be lower in the presence of silane because the latter can convert with HF into fluorosilane and H_2_ [39,50,53]. Consequently, less refluorination takes place, and a higher selectivity towards the formation of the olefin **1** is observed. In C_6_D_6_, the activation of **10a** gave comparable results as when no HSiEt_3_ was introduced (Scheme 4, middle). Compound **10a** is transformed into **1**, **11**, and **12** with the additional presence of traces of the Friedel–Crafts product CF_2_=CFCH_2_C_6_D_5_ (**3**). However, in neat silane, **10a** was converted into **1**, **10b**, **2**, 1,1,1,2-tetrafluoropropane (**13**), 1,1-difluoropropene (**5**), and 1,1,1-trifluoropropane (**6**, Scheme 4, bottom). Thus, by having a large excess of silane, a consecutive reactivity was observed, which led to the formation of the hydrodefluorination product **6** as the main compound. However, the reaction is unselective, and various intermediates are still present in considerable amounts (Scheme 4).

**Scheme 5:**
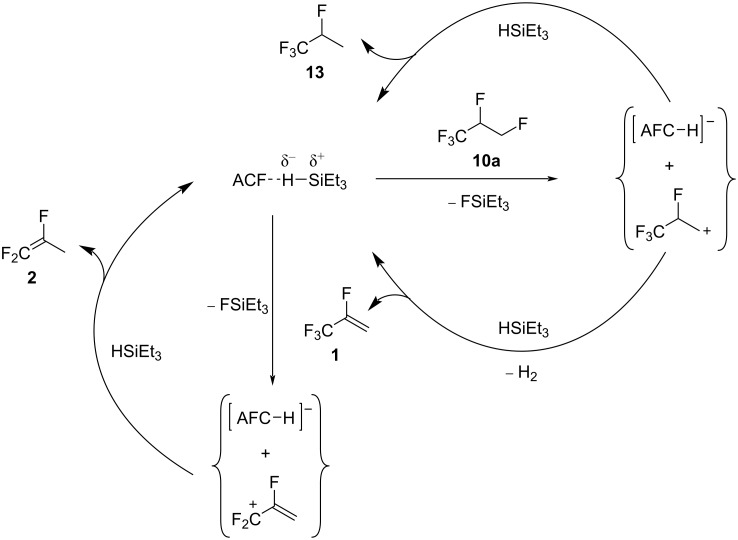
Proposed catalytic cycle for sylilium-mediated hydrodefluorinations and dehydrofluorinations from **10a** at the surface of ACF.

Note, that for the transformation in the presence of silane, the conversions reached over 99% after 7 days at 70 °C, which underlines the significant role of the silane in the reaction mixture. The improved conversion can generally arise from an interaction of silane with the surface of ACF, also competing with the above-mentioned benzene interaction. Furthermore, in the presence of silane, additional mechanistic C–F activation steps have to be considered (Scheme 5). Basically, the immobilization of silane at the Lewis-acidic surface might result in silylium-like species, which subsequently initiate the C–F bond activation at the primary carbon–fluorine bond in **10a**, generating FSiEt_3_, the corresponding carbenium-like species, and a surface-bound hydride. At this stage, either the hydrodefluorination product **13** can be generated, or dehydrofluorination occurs to furnish the olefin **1** and H_2_, both in the presence of silane. Additionally, **1** can further react with any silylium ion species at the surface of ACF, resulting in a C–F bond cleavage at the CF_3_ group, yielding once again a surface hydride and the corresponding carbenium ion. Subsequently, the allylic hydrodefluorination product **2** is formed. Allylic hydrodefluorination reactions were previously observed at ACF. Indeed, in the presence of silane and ACF, the CF_3_ group in tetrafluoropropenes (HFO-1234yf, **1** and HFO-1234ze, **4**) was transformed into an olefinic CF_2_ group (Scheme 1) [16]. Previous MAS NMR studies also gave evidence for the existence of silylium species at an ACF surface [39,47]. In addition, silylium species that are stabilized by weakly coordinating anions can also catalyze hydrodefluorination reactions in a homogeneous phase with silanes as hydrogen source [54–57]. In contrast, silylium-mediated dehydrofluorination reactions have not been found in a homogeneous phase, but germylium ions can promote such reaction pathways [58]. Nevertheless, the formation of the compounds **1**, **10b**, **11**, and **12** can alternatively be initiated by the Lewis acidity of ACF itself, as outlined above without the presence of silane (see Scheme 3). Therefore, as an alternative to the initial formation of the surface silylium ion species at ACF, it is in principle also conceivable that carbenium species can be initially produced by an abstraction of a fluoride ion from a fluorinated group by the surface of the catalyst. Then, carbenium ions can react with silane to yield hydrodefluorination products, or after dehydrofluorination, HF that in the presence of silane, produces FSiEt_3_ and H_2_ [39,50]. Furthermore, any intermediate carbenium species, generated directly at the ACF surface or via interaction with a silylium species, can be engaged in Friedel–Crafts-like reactions to give with C_6_D_6_ **11** or **3**, which is consistent with previous studies [16].

The formation of the products described above (Scheme 4) involves consecutive reaction steps, such as dehydrofluorination, hydrodefluorination, hydrofluorination, allylic defluorination, hydroarylation, and Friedel–Crafts reactions. In the presence of an excess of silane, the dehydrofluorination product **1** and the hydrodefluorination intermediate **13** are generated simultaneously to further lead to **2**, **5**, and **6** as the main compounds. To get further insight, independent reactions were performed to elucidate reaction patterns and to demonstrate the conceivable transformations between certain products, which were observed in the activation of **10a** in the presence of ACF and HSiEt_3_.

**Scheme 6:**
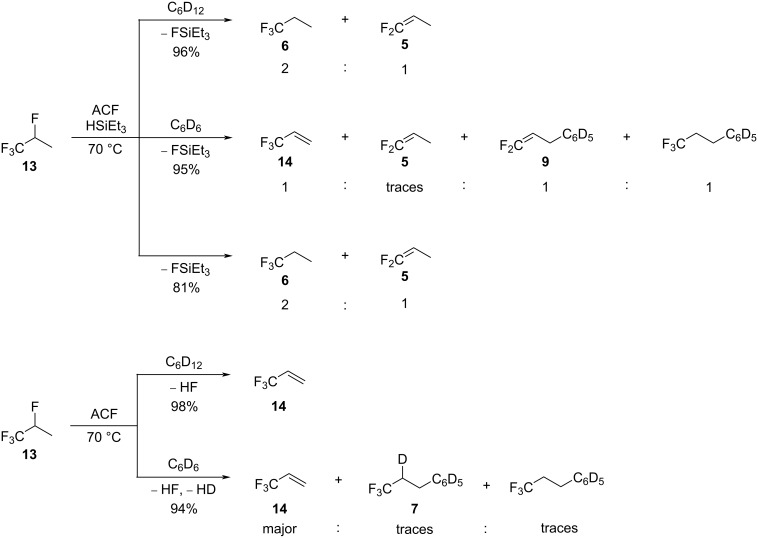
Reactivity of **13** in the presence of ACF as the catalyst, with (top) or without (bottom) HSiEt_3_ as a hydrogen source.

It turned out that the tetrafluoropropene **13** reacts in the presence of silane with ACF as the catalyst in C_6_D_12_ or neat silane to give **5** and **6** (ratio 1:2, Scheme 6, top). When C_6_D_6_ was used as a solvent in the presence of silane, **5**, **14**, **9**, and the Friedel–Crafts product CF_3_CH_2_CH_2_C_6_D_5_ were generated. Note, that in this context, in the absence of silane and in C_6_D_12_, the selective formation of **14** was detected (Scheme 6, bottom). In benzene, **14** was also formed as the main compound, together with the corresponding Friedel–Crafts and hydroarylation products, both observed in traces. This suggests that silane promotes the generation of **5** and **6**, but for the formation of **14** it is not essential.

**Scheme 7:**
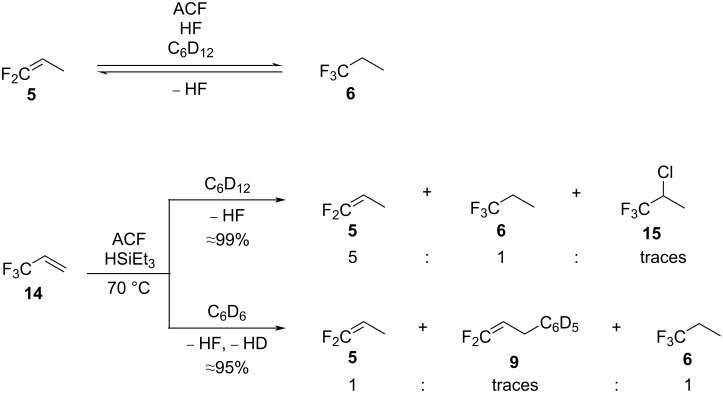
Independent reactions starting from **5**, **6**, or **14** in the presence of ACF as the catalyst.

Furthermore, another independent reaction by treating **5** with HF was performed (Scheme 7, top), but the hydrofluorination product **6** was only detected in small amounts. In accordance with this result, the treatment of **6** in the presence of silane and ACF gave the dehydrofluorination product **5**. The observations nevertheless suggest the presence of an equilibrium between **5** and **6**, that depends on the amount of HF or HSiEt_3_ present in the reaction mixture.

Additionally, it is conceivable that **14** can further react to form **5** via allylic hydrodefluorination [16]. Therefore, the reactivity of **14** was tested in the presence of ACF and silane in C_6_D_12_ or C_6_D_6_ (Scheme 7, bottom). Indeed, in C_6_D_12_, allylic defluorination to yield **5** was observed, together with **6** and traces of 2-chloro-1,1,1-trifluoropropene (**15**). The chlorinated product **15** could stem from a plausible HCl formation if the substrate is fluorinating ACF [41]. It should be noted that the synthesis of ACF itself consists of the fluorination of AlCl_3_ by chlorofluoroalkanes [40–41]. Nevertheless, in C_6_D_6_, the formation of **5** and **6** and the Friedel–Crafts product CF_2_=CHCH_2_C_6_D_5_ (**9**) was observed from **14**.

**Scheme 8:**
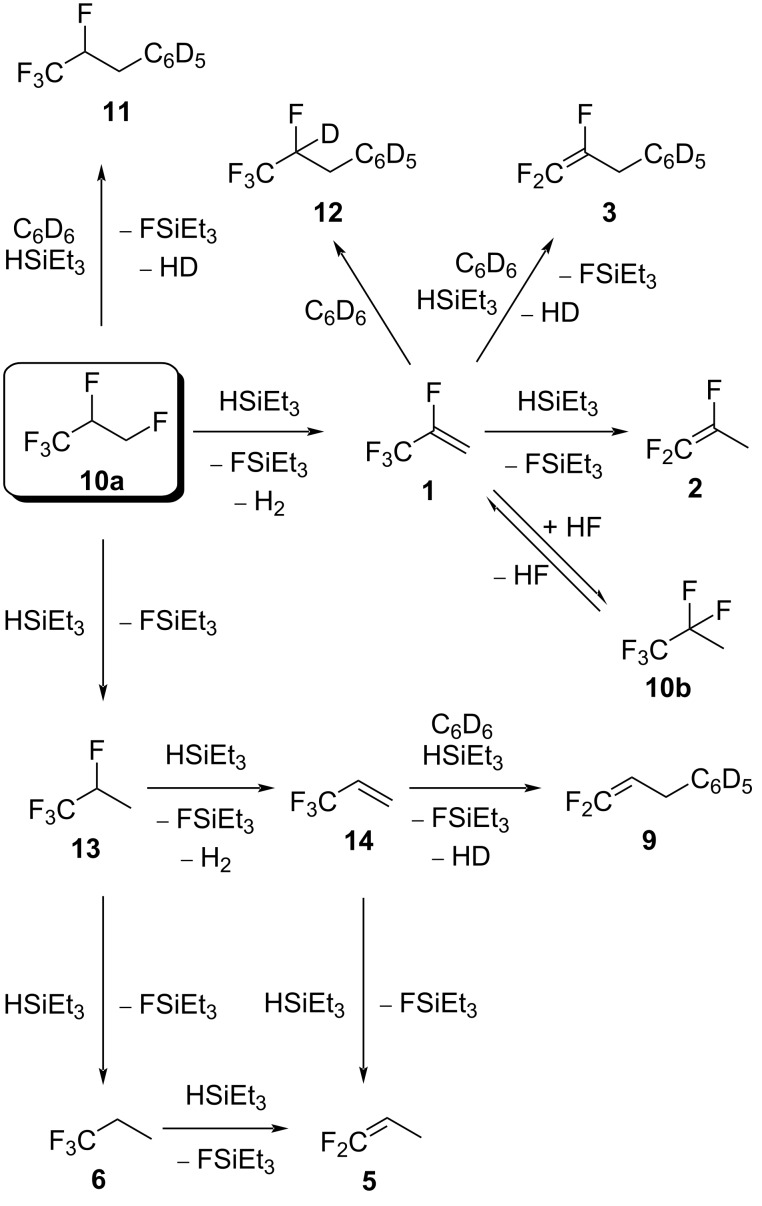
Proposed reaction pathways starting from **10a** in the presence of ACF and silane.

Based on these findings, a general scheme can be drafted to illustrate the sequential generation of products starting from **10a** for the conversions in the presence of silane at ACF (Scheme 8). Note, that the reaction of **1** in the presence of silane, ACF, and solvents was repeated under the same conditions as for the activation of **10a**, and the generation of **2** and **3** was confirmed similarly as reported (see above, Scheme 1) [16].

Overall, the reactivity study on **10a** in the presence of ACF and HSiEt_3_ suggests that the formation of **1** described in the top part of the mechanism is favored when no silane is present in the reaction mixture or when only a small amount of silane is present (Scheme 8). In contrast, in neat silane, the bottom part of Scheme 8 is preferred, leading to the formation of **6**.

### Activation of 1,1,1,3,3-pentafluoropropane (HFC-245fa, **10c**)

The reactivity of 1,1,1,3,3-pentafluoropropane (**10c**) at ACF was compared with the one of the isomer 1,1,1,2,3-pentafluoropropane (**10a**), again to elucidate conceivable reaction pathways and to understand potential similarities in their reactivity. In contrast to the findings for **10a**, no conversion was observed without the use of HSiEt_3_ as a hydrogen source, indicating that for the activation of CHF_2_ groups, silane might be required.

**Scheme 9:**
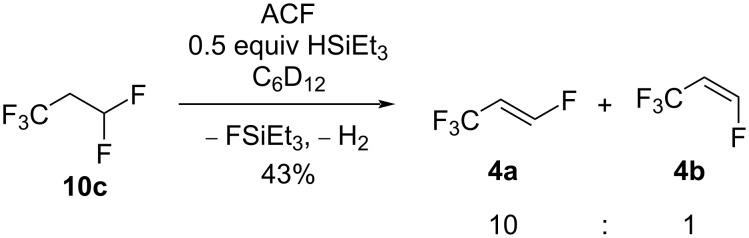
Reactivity of **10c** in the presence of ACF as the catalyst and 0.5 equivalents of HSiEt_3_ as a hydrogen source in C_6_D_12_.

When **10c** was treated with 0.5 equivalents of HSiEt_3_ with respect to the substrate in the presence of ACF at 70 °C, the selective generation of the 1,3,3,3-tetrafluoropropenes (HFO-1234ze, **4a** and **4b**) was detected with an *E:Z* ratio of 10:1 and 43% conversion (Scheme 9). The transformation of **10c** into **4a** and **4b** is remarkable since other catalytic conversions at chromia-based catalysts require elevated temperatures [11,19,59–62].

**Scheme 10:**
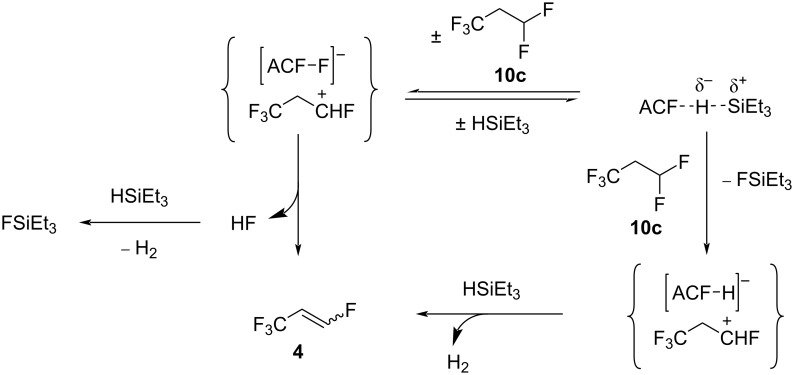
Proposed catalytic cycles for the transformation of **10c** in C_6_D_12_ and in the presence of 0.5 equivalents of silane and ACF as the catalyst.

The mechanisms for the C−F bond cleavage starting from **10c** to yield the isomers **4** are similar to the ones proposed in the activation of **10a** in the presence of silane (see above, Scheme 5). It is likely that silylium species are involved in the C–F activation steps at the ACF surface since HSiEt_3_ is needed to initiate any reactivity. On the one hand, an initial C–F bond activation at **10c** by some silylium ion species will produce FSiEt_3_ and the corresponding carbenium species (Scheme 10). The latter can generate **4a** and **4b** together with H_2_, leading to the regeneration of the catalyst. When only 0.5 equivalents of silane are present, the reaction does not pursue further, as shown above (Scheme 9). On the other hand, as for **10a**, an alternative mechanism can be proposed where the carbenium species is initially generated by an abstraction of a fluoride ion at the CHF_2_ group by the surface of the catalyst. Via HF elimination, **4a** and **4b** are produced, and the conversion is driven by the HF reaction with HSiEt_3_ to give FSiEt_3_.

**Scheme 11:**
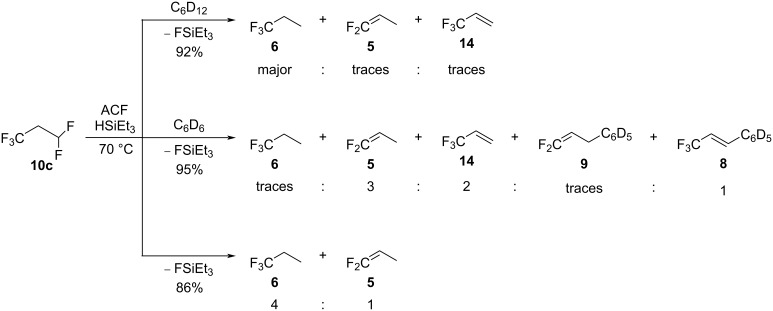
Reactivity of **10c** in the presence of ACF as the catalyst and HSiEt_3_ as a hydrogen source in C_6_D_12_ (top) or C_6_D_6_ (middle) as solvents or in neat silane (bottom).

When the amount of silane was increased to one equivalent, further reactivity was observed (Scheme 11). In C_6_D_12_, **6** was generated as the main compound, with traces of **5** and **14** (Scheme 11, top). When C_6_D_6_ was used as the solvent, **5** as the main product, **6** as a minor product, and **14** in traces were again generated, together with the Friedel–Crafts products CF_2_=CHCH_2_C_6_D_5_ (**9**) and CF_3_CH=CHC_6_D_5_ (**8**, Scheme 11, middle). In neat silane, **5** and **6** were detected in a ratio of 1:4 (Scheme 11, bottom).

Notably, for all conversions (C_6_D_6_ and silane, C_6_D_12_ and silane, or in neat silane), monitoring of the reaction by ^19^F NMR spectroscopy led to the observation of the early generation of **4a** and **4b** after 24 hours reaction time at 70 °C. After 3 days at 70 °C, the transformation into the different products described above could be detected. Moreover, the continuous formation of FSiEt_3_ over time was observed for all reactions, which underlines the crucial role of the hydrogen source at each reaction step. As stated, it has been reported before that for the activation of 1,3,3,3-tetrafluoropropene (HFO-1234ze, **4**) at ACF in neat HSiEt_3_ 1,1-difluoropropene (**5**) and 1,1,1-trifluoropropane (**6**) are furnished (Scheme 1) [16]. In the presence of C_6_D_6_ and silane, the Friedel–Crafts products CF_2_=CHCH_2_C_6_D_5_ (**9**) and CF_3_CH=CHC_6_D_5_ (**8**) were observed, together with the hydroarylation product CF_3_CHDCH_2_C_6_D_5_ (**7**) [16]. To note, in neat silane, a distinct selectivity was detected, depending on the substrates used. Indeed, starting from **10c**, the product ratio between **5** and **6** was 1:4, whereas it was reported to be 1:0.8 starting from **4a** [16]. This observation might be the result of a hydrofluorination reaction from **5** to **6**. Moreover, in neat silane, **14** was not detected, whereas when less silane and a solvent was present, this product was observed. Due to a large amount of silane present, a more considerable amount of the silylium species can be generated compared to when only one equivalent of silane is used. Therefore, the rate of the reaction from **10c** to **6** might be increased in neat silane favoring two subsequent hydrodefluorination steps from **10c** to yield **6**.

**Scheme 12:**
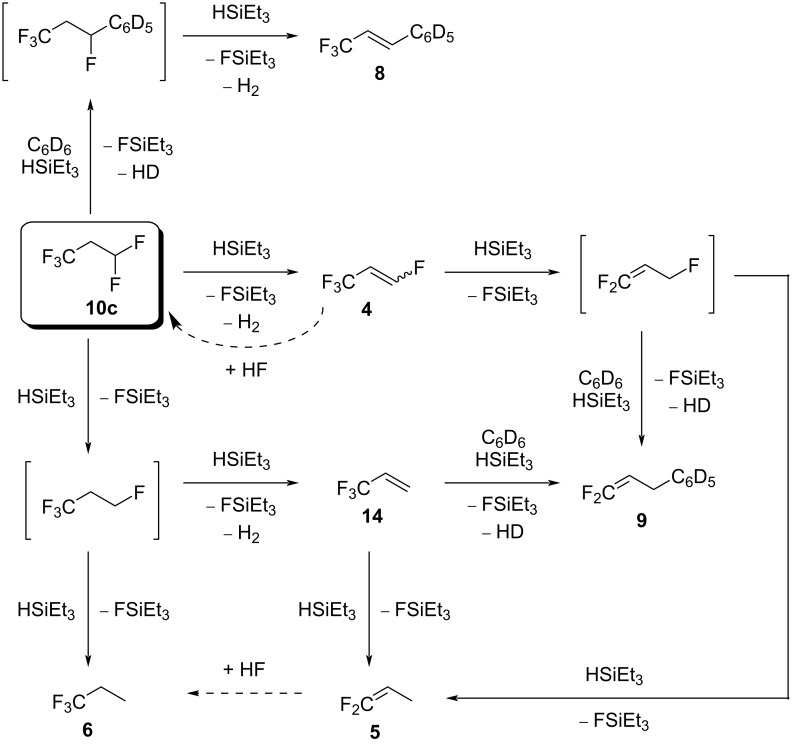
Proposed reaction pathways starting from **10c** in the presence of ACF and silane.

As observed in the study on the reactivity of **10a**, the variety of products for a larger amount of silane can be explained by several consecutive reactions, which include C–F bond activation steps (Scheme 12). Via hydrodefluorination, **10c** can produce the intermediate 1,1,1,3-tetrafluoropropane (not observed). This intermediate could give **6** via a second hydrodefluorination. Alternatively, 1,1,1,3-tetrafluoropropane could also produce **14**, which was shown to give **6** via the intermediate **5** by independent reactions. However, pathways to yield **6** are also required from the olefins **4**, because they were detected as intermediates. This implies HF addition to **4** or **5**. In the presence of benzene, **14** can also generate the Friedel–Crafts product **9**; however, product **9** can also be generated from **4** by hydrodefluorination followed by a Friedel–Crafts reaction. The Friedel–Crafts product **8** can as well be formed from **10c** directly, via a Friedel–Crafts reaction followed by HF elimination.

Comparable to the reaction patterns of **10a**, the reactivity study on **10c** reveals that with a small amount of silane, the dehydrofluorination to **4** is favored, and with more silane, the conversions in Scheme 12 end up favorably in the formation of **6**.

### Activation of 1,1,1,2,2-pentafluoropropane (HFC-245fa, **10b**)

**Scheme 13:**
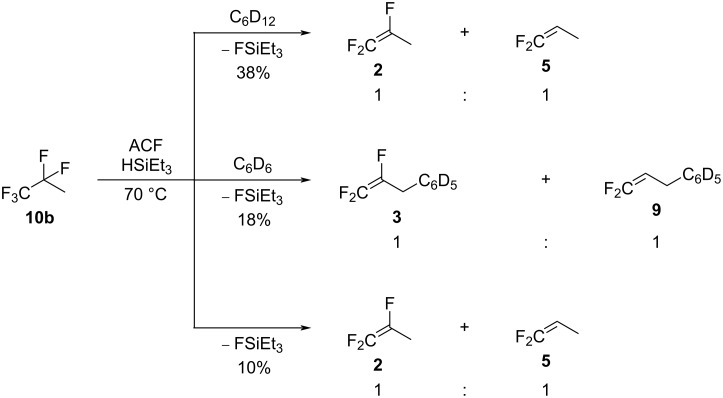
Reactivity of **10b** in the presence of ACF as the catalyst and HSiEt_3_ as a hydrogen source in C_6_D_12_ (top) or C_6_D_6_ (middle) as solvents or in neat silane (bottom).

As observed for **10c**, the isomeric 1,1,1,2,2-pentafluoropropane (HFC-245fa, **10b**) could also not be activated without the presence of HSiEt_3_ as a hydrogen source. The treatment of **10b** in C_6_D_12_ in the presence of silane at 70 °C gave the allylic hydrodefluorination product **2** as well as **5** in a ratio of 1:1 with 38% conversion (Scheme 13, top). When benzene was used as a solvent together with silane, the Friedel–Crafts products **3** and **9** were observed in a ratio of 1:1, but the conversion reached only 18% (Scheme 13, middle). In neat silane, **2** and **5** were again observed, but this time the conversion did not exceed 10% (Scheme 13, bottom).

As for the activation of **10c**, it is plausible that surface silylium ion species are formed at ACF. Therefore, a reaction pathway can be suggested, starting with the initial generation of **1**, FSiEt_3_, and H_2_. Subsequently, **2** can be generated via an allylic hydrodefluorination as it was observed in the study of tetrafluoropropenes at ACF [16]. Additionally, **5** can stem from several C–F bond activations at **10b**, starting with a hydrodefluorination to generate the intermediate **13**, which further undergoes an HF elimination, followed by an allylic hydrodefluorination to give **5**. In the presence of benzene, at **1**, a Friedel–Crafts reaction can generate **3**, which further supports the formation as an intermediate of **1**. Moreover, the intermediate **14** can also produce **9** via a Friedel–Crafts reaction, which again supports the pathway proposed to achieve **5**.

**Scheme 14:**
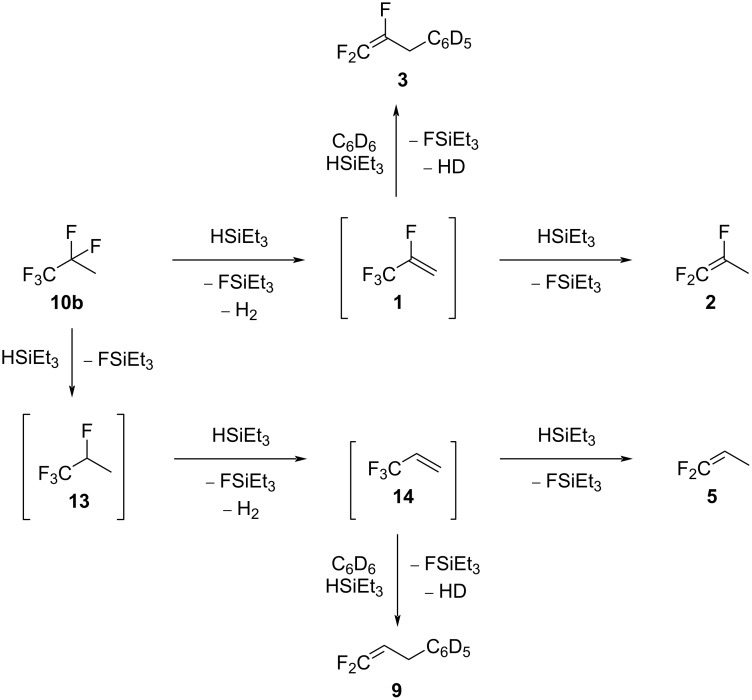
Proposed reaction pathway starting from **10b** in the presence of ACF and silane.

Overall, for the reactivity of the pentafluoropropane isomer **10b**, two different pathways seem to compete. The upper part of the reaction patterns in Scheme 14 leads to the formation of **2**, and the bottom part provides pathways to **5**. Thus, the hydrodefluorination step at **10b** to form **13** seems to be more difficult than for the other isomers **10a** and **10c**.

## Conclusion

This study on the reactivity of pentafluoropropane isomers has revealed that ACF is a suitable catalyst for dehydrofluorination and hydrodefluorination reactions of polyfluorinated compounds under mild conditions. It was elaborated that the single C–F bond in **10a** does not require the use of HSiEt_3_ as a hydrogen source to be activated via dehydrofluorination. However, when silane was introduced, further reactivity was observed, leading to the formation of subsequent defluorination products. In contrast, CF_2_, CHF_2_, and CF_3_ groups need the presence of a hydrogen source in order to promote the activation of at least one C–F bond by the formation of the thermodynamically stable Si–F bond. This observation is consistent with a decrease of the bond dissociation energies of C–F bonds from trifluoromethyl via difluoromethyl to monofluoromethyl groups [63]. Additionally, the C–F bond at the CHF_2_ group in **10c** was easier to activate than the C–F bond in the CF_3_CF_2_ group in **10c**, resulting in an order of reactivity of **10a** > **10c** > **10b**. When only a small amount of silane was introduced for the reaction of **10a** or **10c**, the major products are due to dehydrofluorination, whereas in neat silane, formally hydrodefluorination products are generated mainly. By using C_6_D_6_, various Friedel–Crafts products can be further generated. For **10b**, the conversions are very low, but dehydrofluorination and hydrodefluorination pathways compete with each other.

Mechanistically, the C–F bonds of the fluorinated substrates can be activated by Lewis-acidic sites at the ACF surface. In the presence of silane, it can be assumed that preferentially silylium surface species initiate the C–F bond cleavage. For both, the generated carbenium species show further reactivity to result in dehydrofluorination, hydrodefluorination, or Friedel–Crafts products. Notably, the conversion in neat silane was lower in the case of **10c** and **10b**, possibly because of a certain blocking of the acidic sites of ACF by silane. Note in that context that there are reports showing that silylium species can interact with more silane to generate larger entities [56,58,64].

## Experimental

### Material and methods

The reactions were carried out using Schlenk techniques as well as JYoung NMR tubes. The solvents were purchased from Eurisotop. C_6_D_12_ was dried over molecular sieves and purged with argon prior to use. C_6_D_6_ was dried with K-Solvona and distilled prior to use. Et_3_SiH (99%) was purchased from Sigma–Aldrich in a sure seal bottle and stored under argon. 1,1,1,2,3-Pentafluoropropane (HFC-245eb, **10a)**, 1,1,1,3,3-pentafluoropropane (HFC-245fa, **10c**), and 1,1,1,2,2-pentafluoropropane (HFC-245cb, **10b**) were gifted by Arkema and used without further purification. 3,3,3-Trifluoropropene (**14**, 99%), 1,1,1-trifluoropropane (**6**), and 1,1,1,2-tetrafluoropropane (**13**) were purchased from abcr and used without further purification. 1,1-Difluoropropene (**5**) was bought from Apollo Scientific and used without further purification. ACF was synthesized according to the literature and stored in a glove box. The number of active sites (1 mmol acidic sites/g of catalyst) was determined by temperature programmed desorption of ammonia (NH_3_-TPD) [34,65]. NMR spectra were recorded at room temperature using a Bruker DPX 300 spectrometer. A capillary of trifluorotoluene was employed as an external standard for quantification purposes. The ^19^F NMR spectra were referenced to PhCF_3_ (δ = −63.5 ppm) and the chemical shifts in ^1^H NMR were referenced to residual C_6_D_5_H (δ = 7.16 ppm) or C_6_D_11_H (δ = 1.38 ppm).

### Procedure for reactions with gaseous substrates

A JYoung NMR tube was loaded with 25 mg of ACF inside a glovebox. In experiments involving a solvent (C_6_D_6_ or C_6_D_12_), 0.4 mL of the solvent was added under Schlenk conditions, together with the corresponding amount of HSiEt_3_. In the reactions without solvent, 0.5 mL of HSiEt_3_ was added using Schlenk techniques to the JYoung NMR tube loaded with ACF. The gases were then condensed using a small glass bulb filled with 0.5 atm of the corresponding gas (0.1 mmol). The reactions were monitored by ^1^H and ^19^F NMR spectroscopy. The tubes were kept at 70 °C for 7 days. PhCF_3_ was used as an external standard in a closed capillary to calculate the conversion based on the consumed substrate by the integration of the ^19^F NMR spectra.

## Supporting Information

File 1 Analytical data and copies of spectra.
