# Supplementary material for: Activation of pentafluoropropane isomers at a nanoscopic aluminum chlorofluoride: hydrodefluorination versus dehydrofluorination
Source: Beilstein J Org Chem. 2020 Oct 23;16:2623–35. doi: 10.3762/bjoc.16.213 (PMC7590627; doi:10.3762/bjoc.16.213)
Supplement: File 1 — Analytical data and copies of spectra. [file Beilstein_J_Org_Chem-16-2623-s001.pdf]

BEILSTEIN JOURNAL OF ORGANIC CHEMISTRY

Supporting Information

for

Activation of pentafluoropropane isomers at a nanoscopic aluminum chlorofluoride: hydrodefluorination versus dehydrofluorination

Maëva-Charlotte Kervarec, Thomas Braun, Mike Ahrens and Erhard Kemnitz

Beilstein J. Org. Chem. **2020,** *16,* 2623–2635. doi:10.3762/bjoc.16.213

Analytical data and copies of spectra

License and Terms: This is a supporting information file under the terms of the Creative Commons Attribution License (https://creativecommons.org/licenses/by/4.0). Please note that the reuse, redistribution and reproduction in particular requires that the authors and source are credited.
The license is subject to the *Beilstein Journal of Organic Chemistry* terms and conditions: (https://www.beilstein-journals.org/bjoc)

1- General procedure

In a JYoung NMR tube, 25 mg of ACF was suspended in either C_6D_6 or C_6D_{12} (0.4 mL) as solvent. In the reactions involving a silane, either 60 µL of silane were added to the tubes containing a solvent (0.4 mL) or the reactions were performed in neat silane (0.5 mL). The gases were then condensed using a small glass bulb filled with 0.5 atm of the corresponding gas (0.1 mmol). The reactions were monitored by ^{1}H and ^{19}F NMR spectroscopy over 7 days and the tubes were kept at 70 °C. $PhCF_3$ or C_6F_6 was used as an external standard in a closed capillary in order to calculate the conversion based on the consumed substrate by the integration of ^{19}F NMR spectra.

2- Activation of 1,1,1,2,3-pentafluoropropane **10a**

a. Without the presence of silane

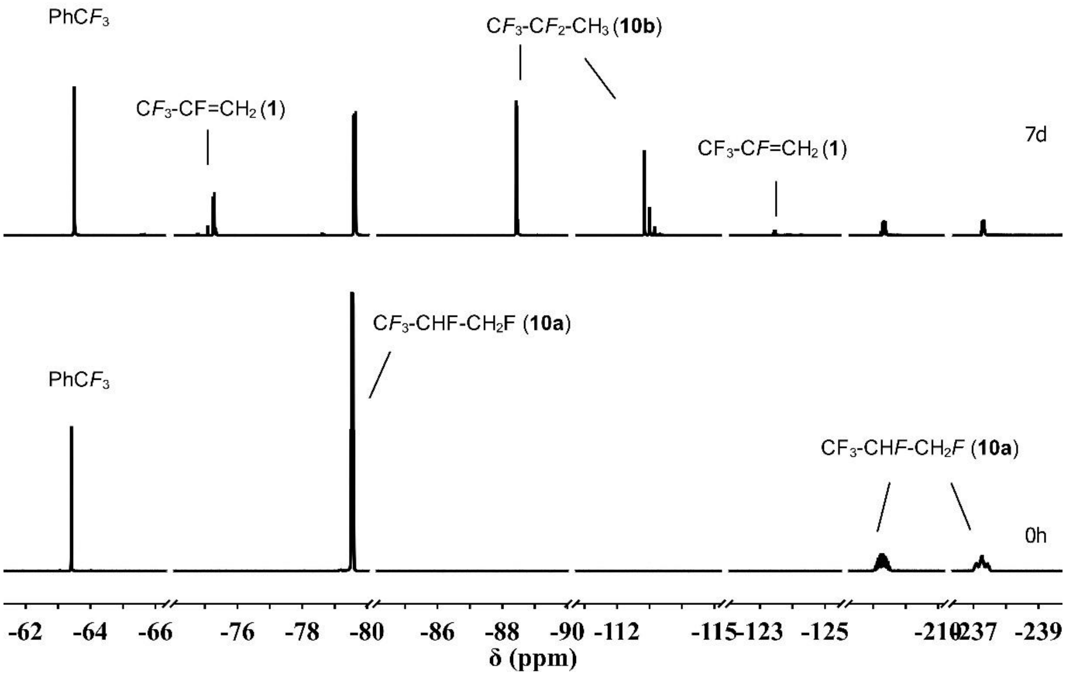

Figure 1: ^{19}F NMR spectra for the activation of **10a** in the presence of ACF as the catalyst in C_6D_{12}.

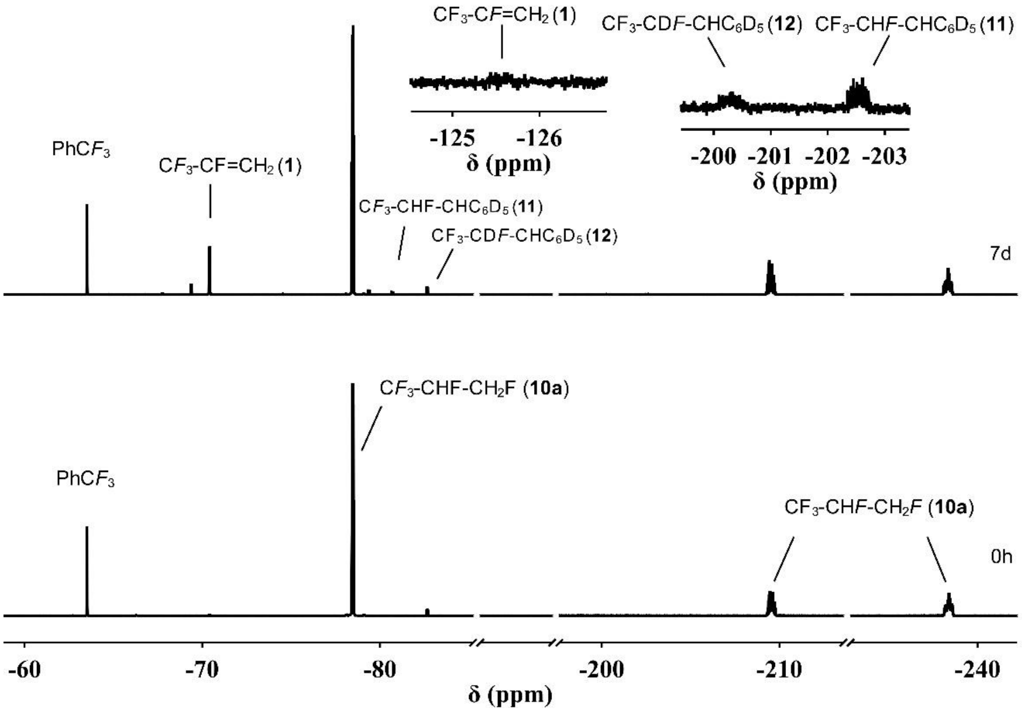

Figure 2: ^{19}F NMR spectra for the activation of **10a** in the presence of ACF as the catalyst in C_6D_6.

b. In the presence of silane

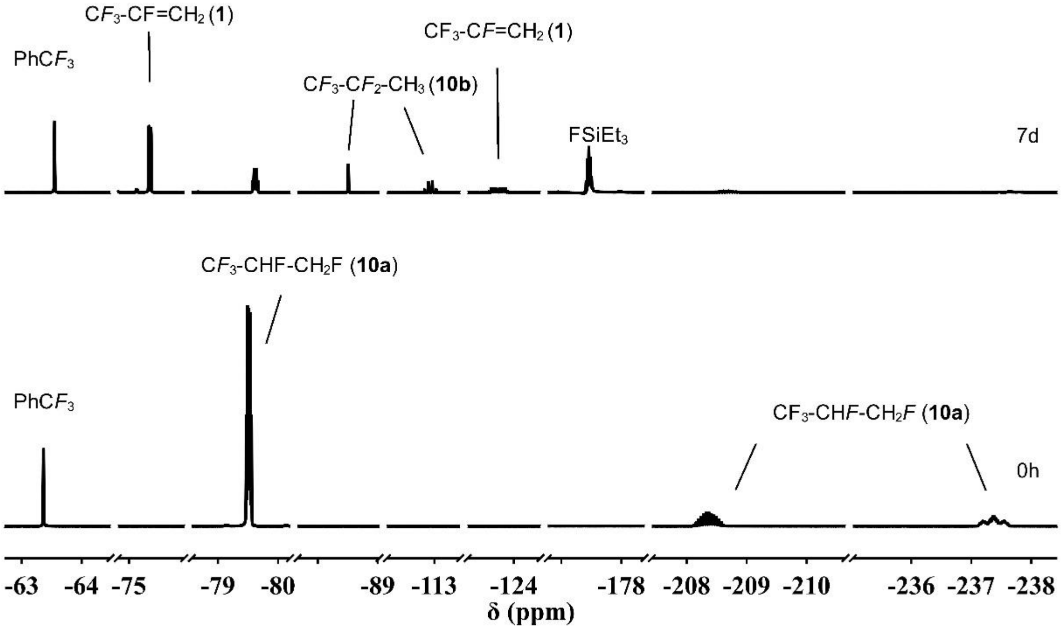

Figure 3: ^{19}F NMR spectra for the activation of **10a** in the presence of ACF as the catalyst and $HSiEt_3$ as the hydrogen source in C_6D_{12}.

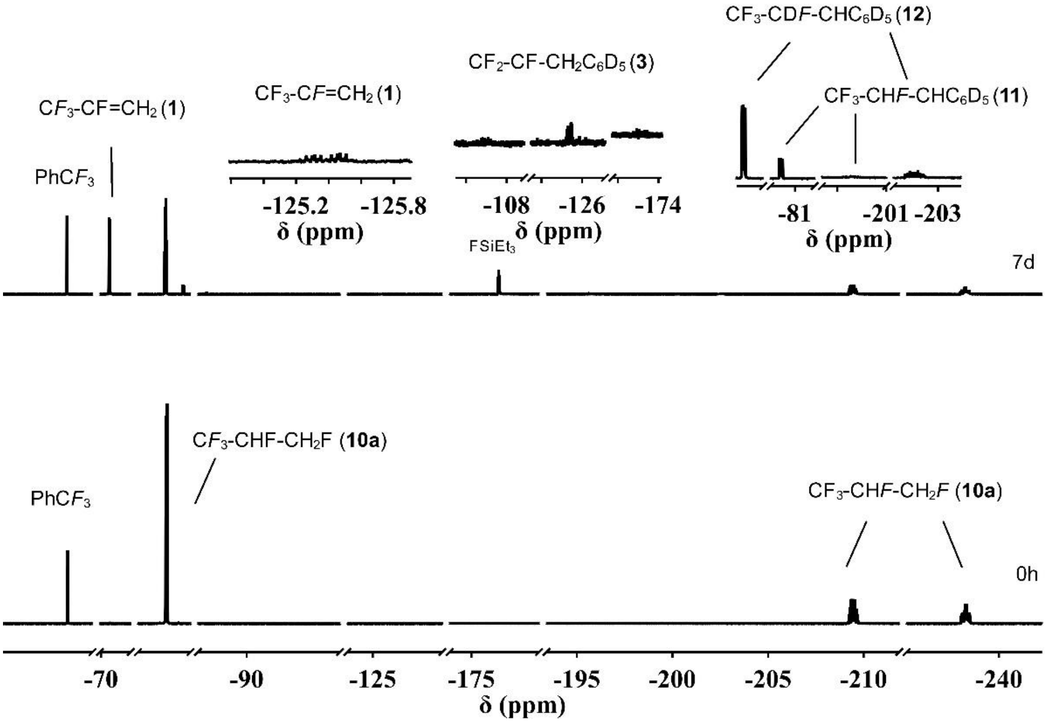

Figure 4: ^{19}F NMR spectra for the activation of **10a** in the presence of ACF as the catalyst and $HSiEt_3$ as the hydrogen source in C_6D_6.

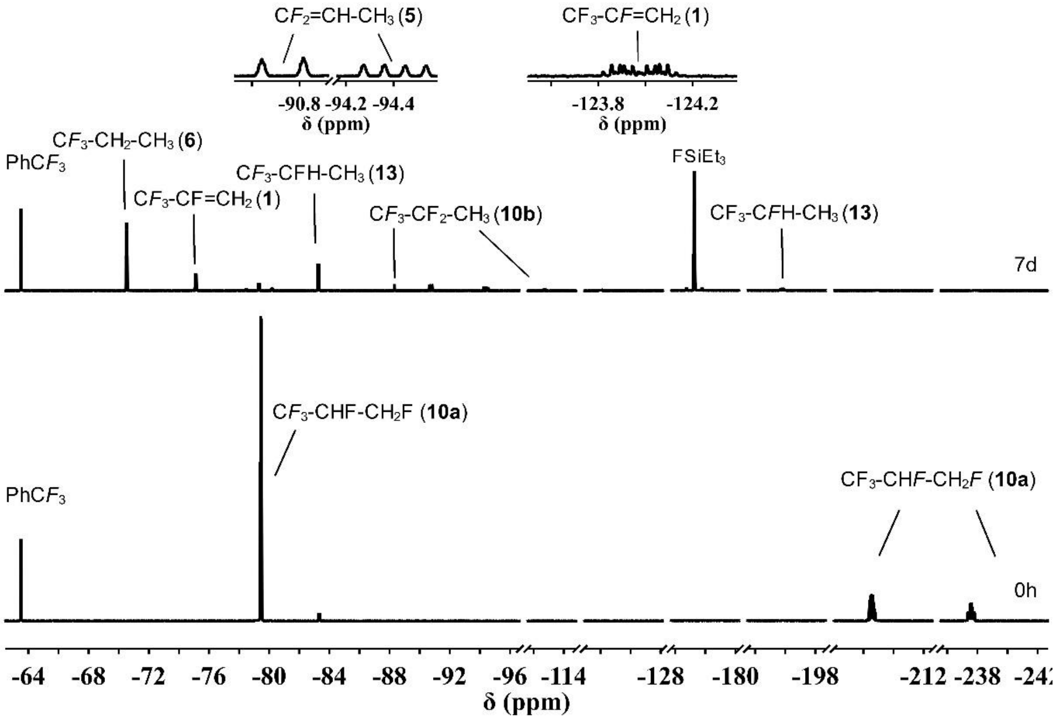

Figure 5: ^{19}F NMR spectra for the activation of **10a** in the presence of ACF as the catalyst and $HSiEt_3$ as the hydrogen source (neat).

3- Activation of 1,1,1,3,3-pentafluoropropane **10c**

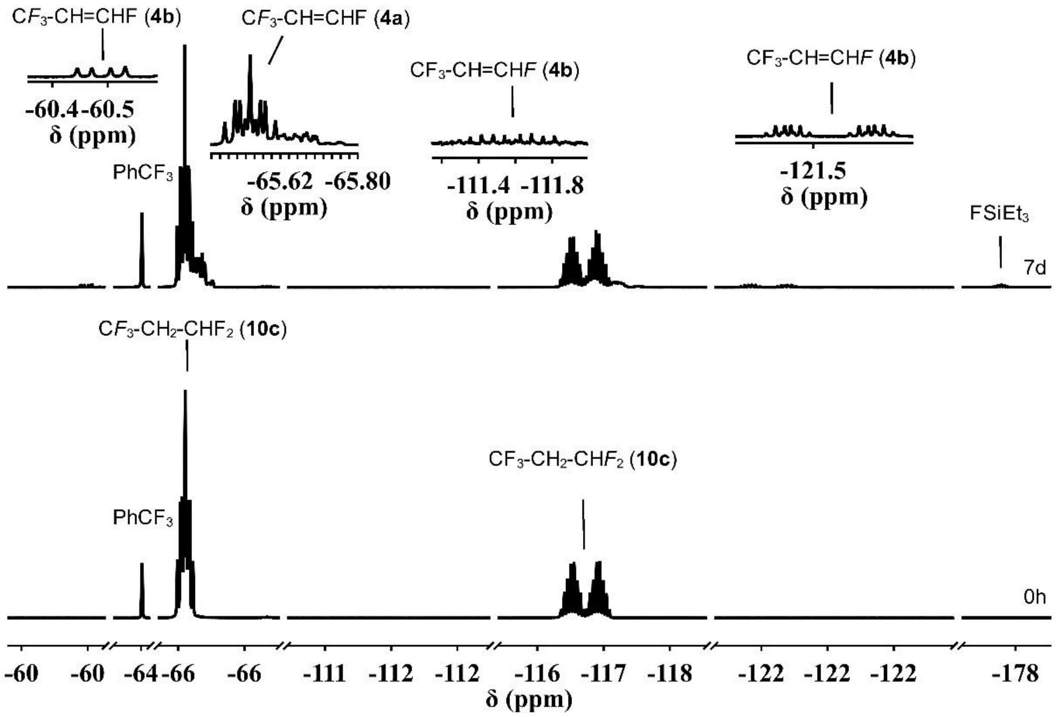

Figure 6: ^{19}F NMR spectra for the activation of **10c** in the presence of ACF as the catalyst and 0.5 equivalent of $HSiEt_3$ as the hydrogen source in C_6D_{12}.

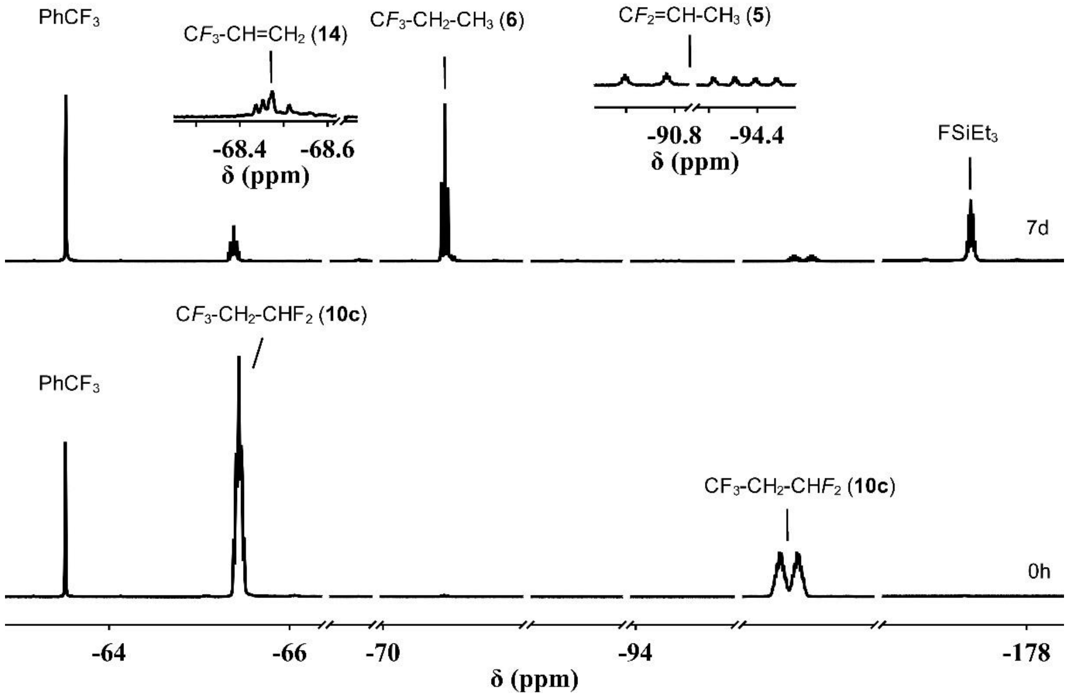

Figure 7: ^{19}F NMR spectra for the activation of **10c** in the presence of ACF as the catalyst and $HSiEt_3$ as the hydrogen source in C_6D_{12}.

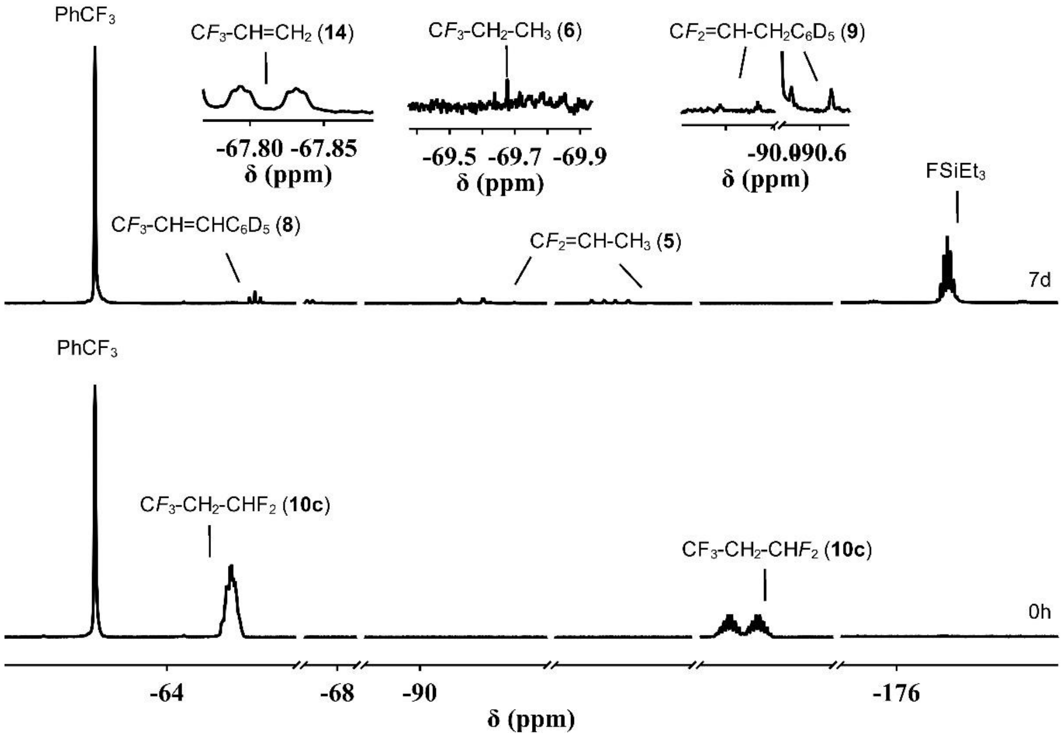

Figure 8: ^{19}F NMR spectra for the activation of **10c** in the presence of ACF as the catalyst and $HSiEt_3$ as the hydrogen source in C_6D_6.

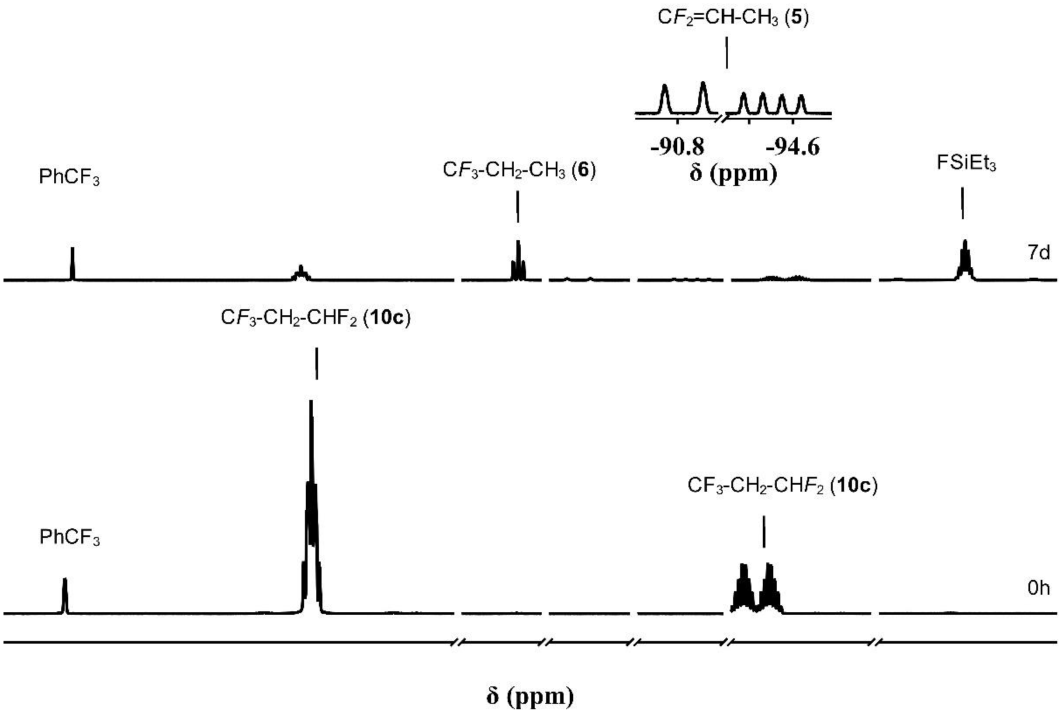

Figure 9: ^{19}F NMR spectra for the activation of **10c** in the presence of ACF as the catalyst and $HSiEt_3$ as the hydrogen source (neat).

4- Activation of 1,1,1,2,2-pentafluoropropane **10b**

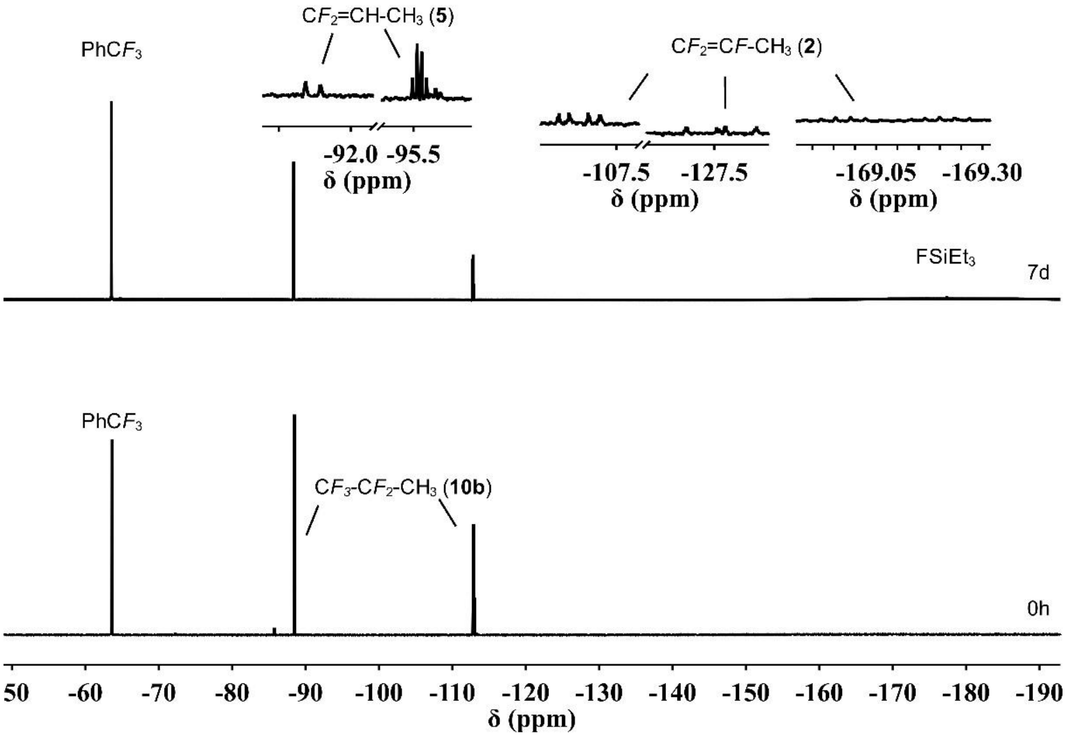

Figure 10: ^{19}F NMR spectra for the activation of **10b** in the presence of ACF as the catalyst and $HSiEt_3$ as the hydrogen source in C_6D_{12}.

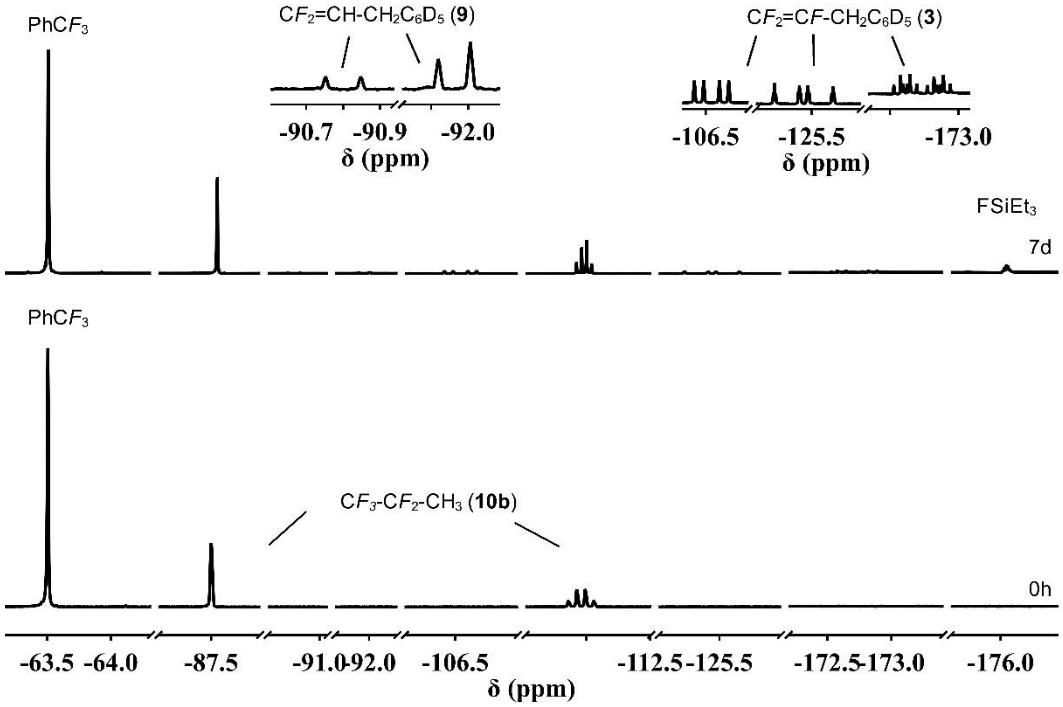

Figure 11: ^{19}F NMR spectra for the activation of **10b** in the presence of ACF as the catalyst and $HSiEt_3$ as the hydrogen source in C_6D_6.

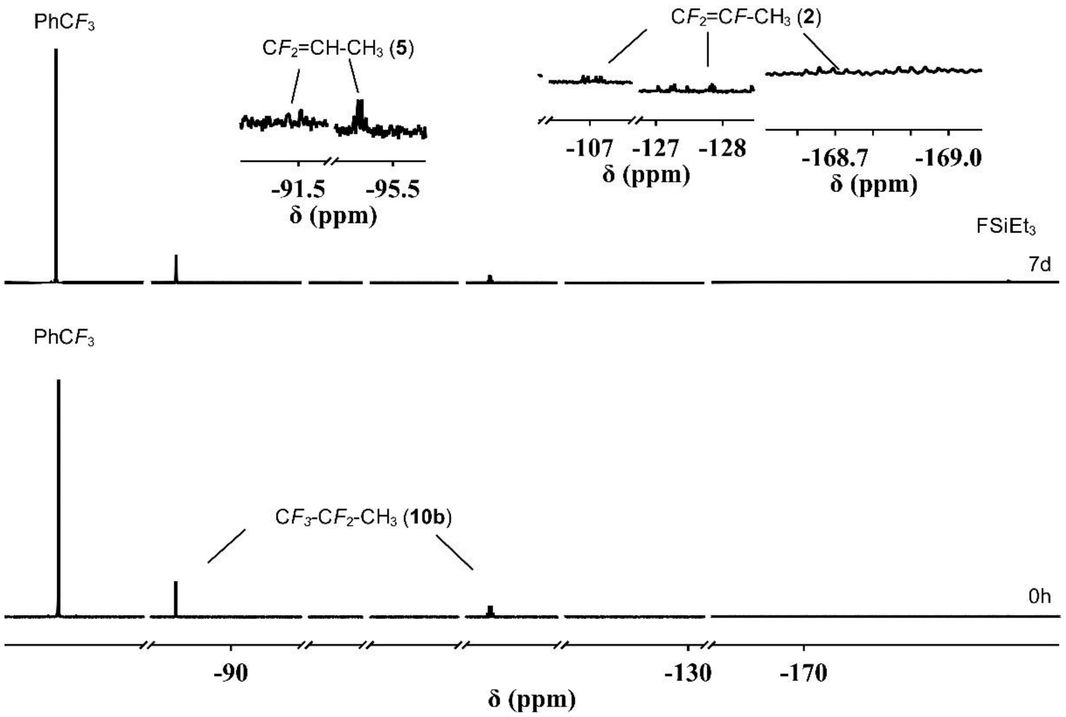

Figure 12: ^{19}F NMR spectra for the activation of **10b** in the presence of ACF as the catalyst and $HSiEt_3$ as the hydrogen source (neat).

5- Independent reactions

c. 1,1,1,2-tetrafluoropropane **13**

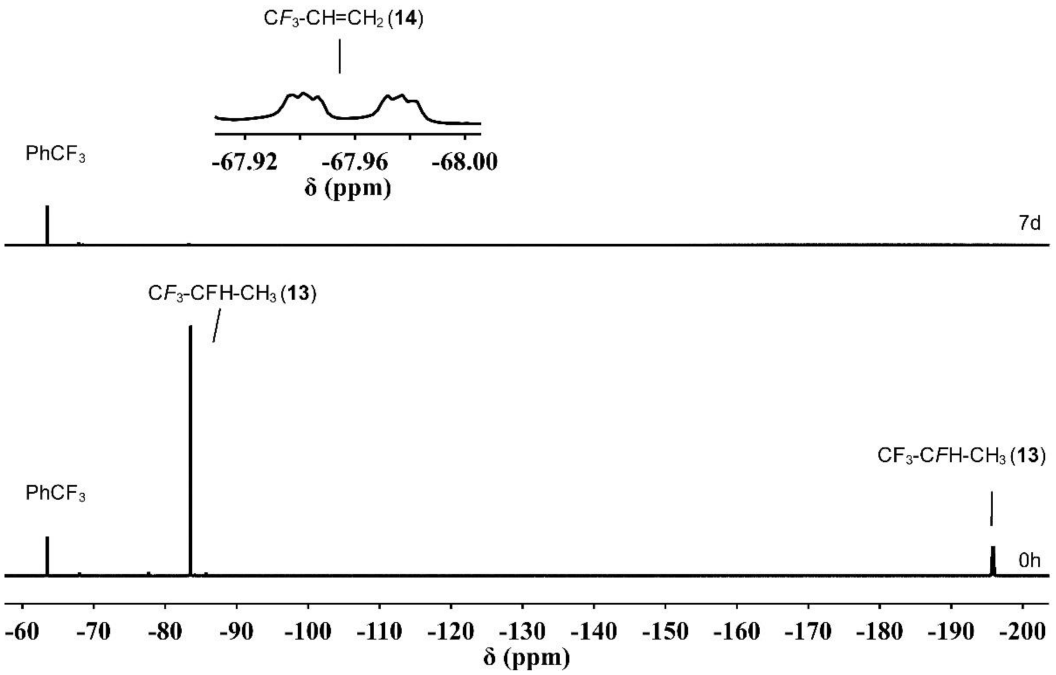

Figure 13: ^{19}F NMR spectra for the activation of **13** in the presence of ACF as the catalyst in C_6D_{12}.

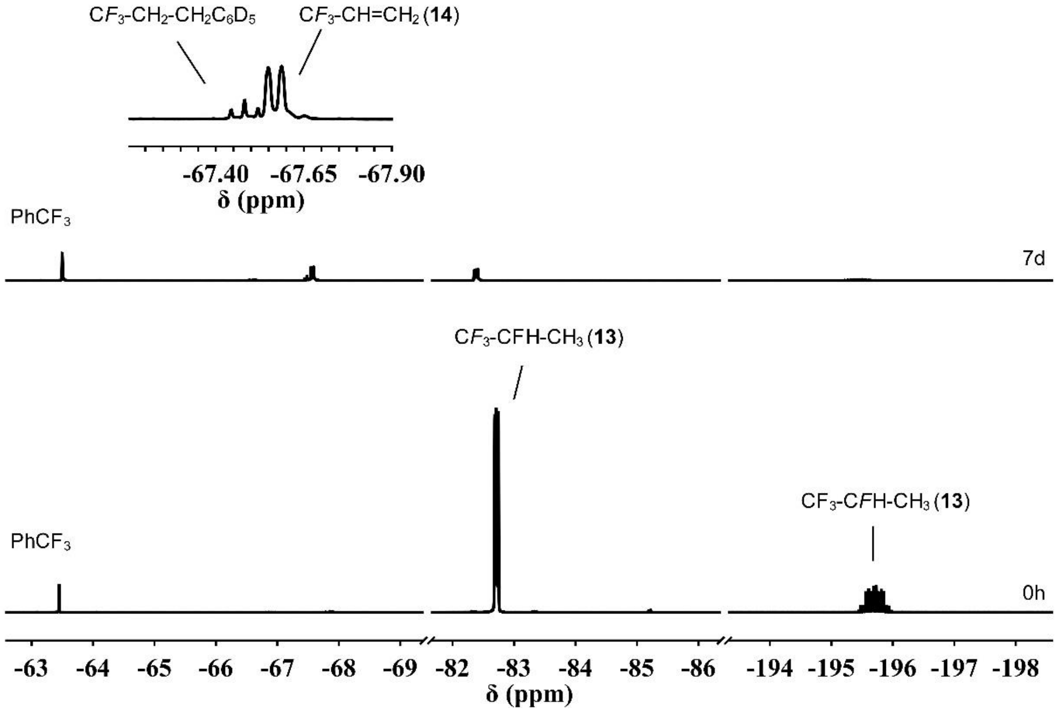

Figure 14: ^{19}F NMR spectra for the activation of **13** in the presence of ACF as the catalyst in C_6D_6.

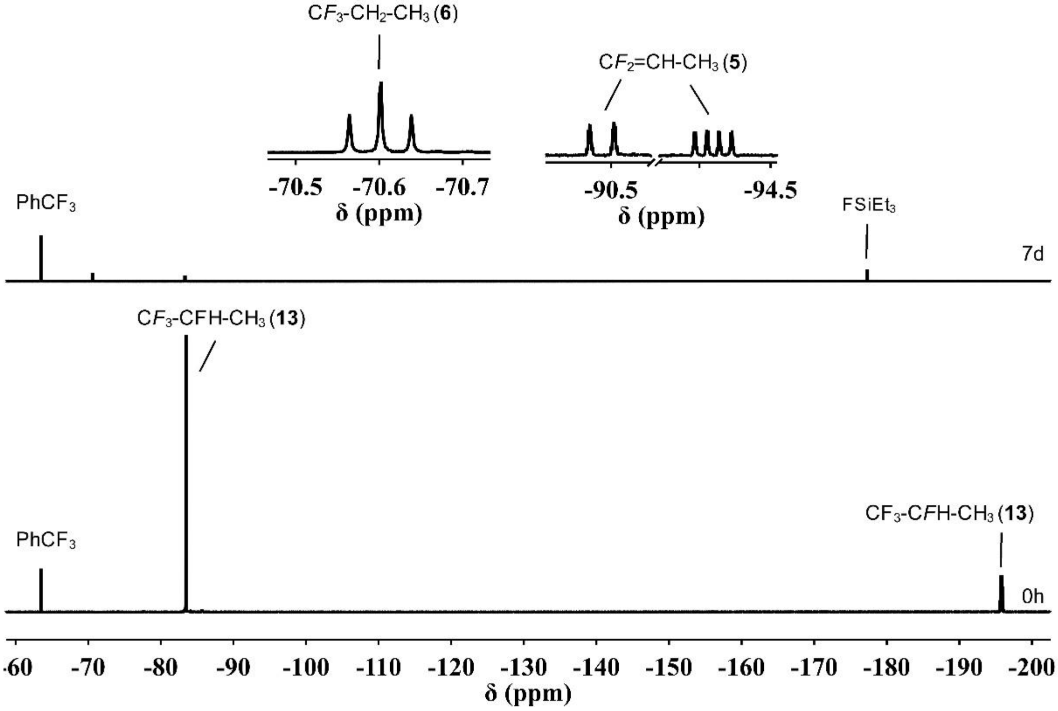

Figure 15: ^{19}F NMR spectra for the activation of **13** in the presence of ACF as the catalyst and $HSiEt_3$ as the hydrogen source in C_6D_{12}.

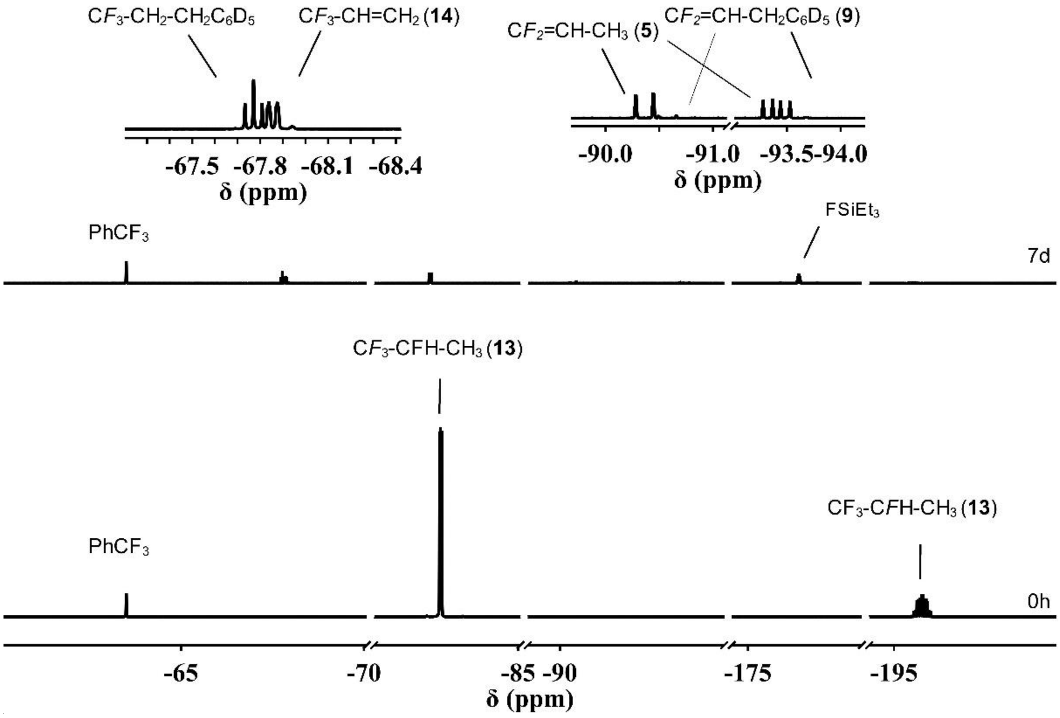

Figure 16: ^{19}F NMR spectra for the activation of **13** in the presence of ACF as the catalyst and $HSiEt_3$ as the hydrogen source in C_6D_6.

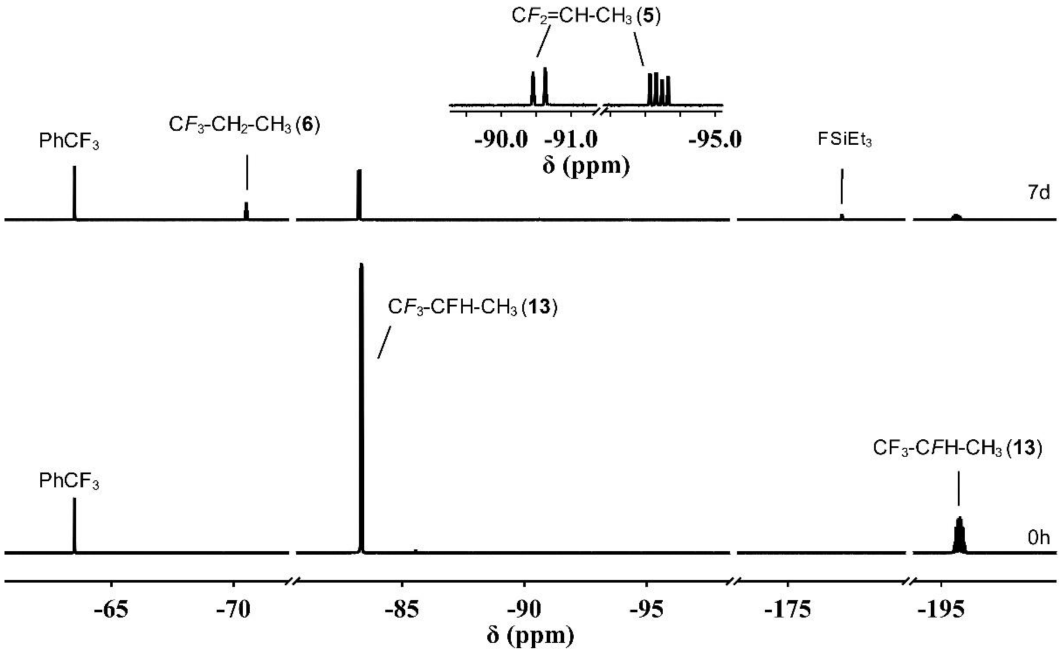

Figure 17: ^{19}F NMR spectra for the activation of **13** in the presence of ACF as the catalyst and $HSiEt_3$ as the hydrogen source (neat).

d. 1,1-difluoropropene **5**

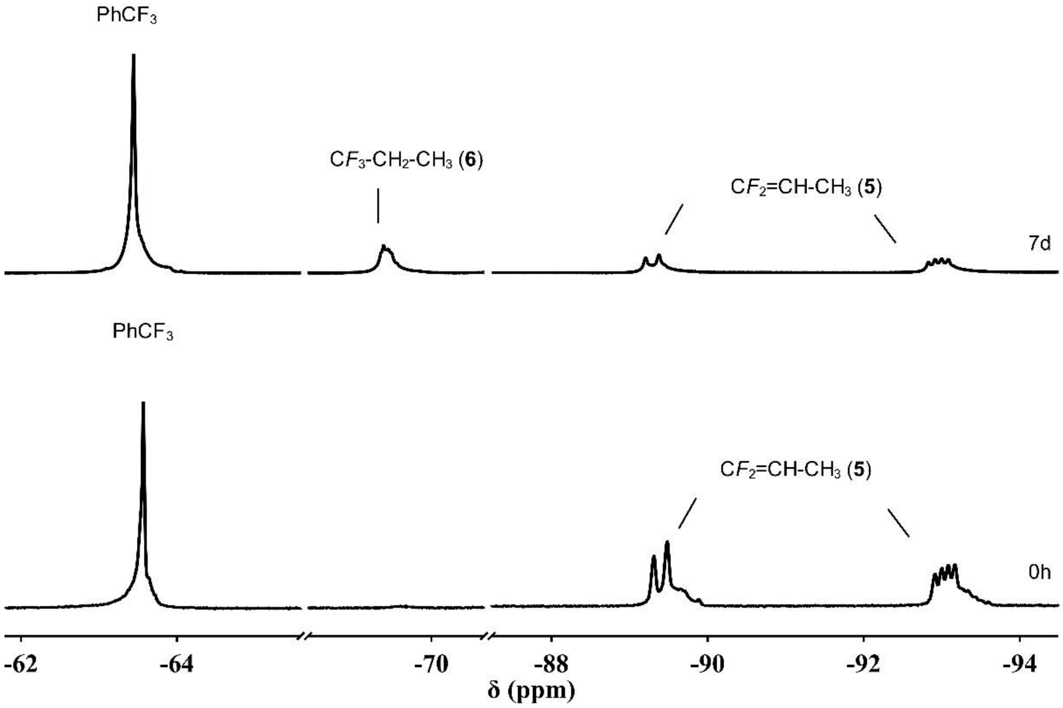

Figure 18: ^{19}F NMR spectra for the activation of **5** in the presence of ACF as the catalyst and HF in C_6D_{12}.

e. 1,1,1-trifluoropropane **6**

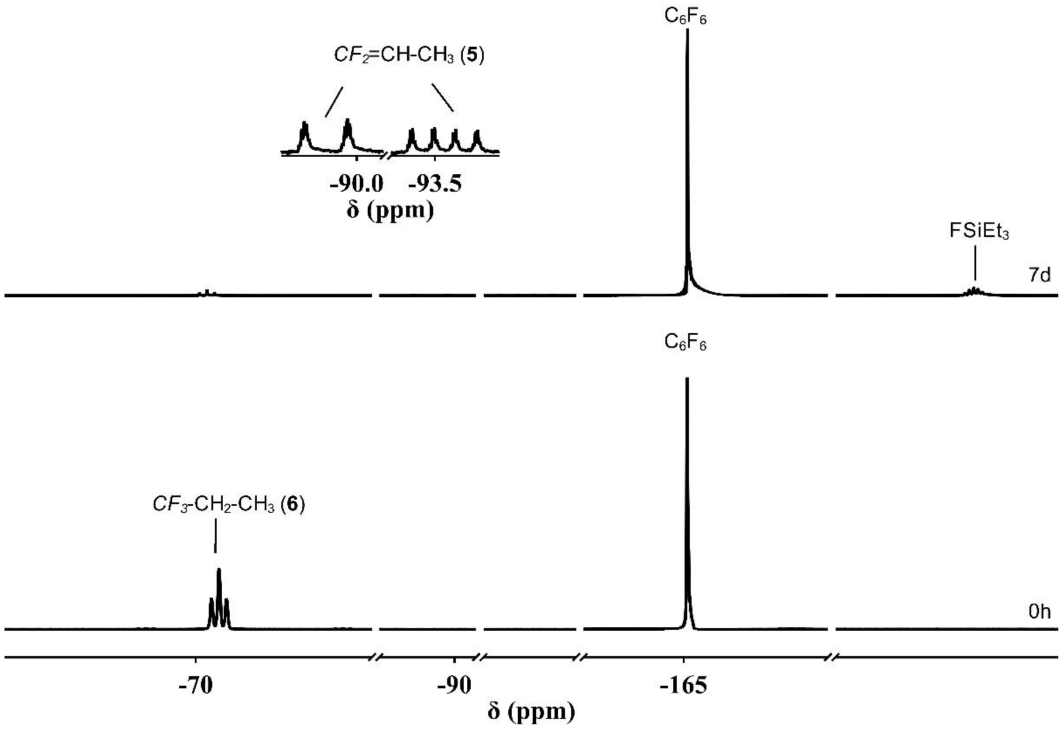

Figure 19: ^{19}F NMR spectra for the activation of **6** in the presence of ACF as the catalyst and $HSiEt_3$ as the hydrogen source in C_6D_{12}.

f. 3,3,3-trifluoropropene **14**

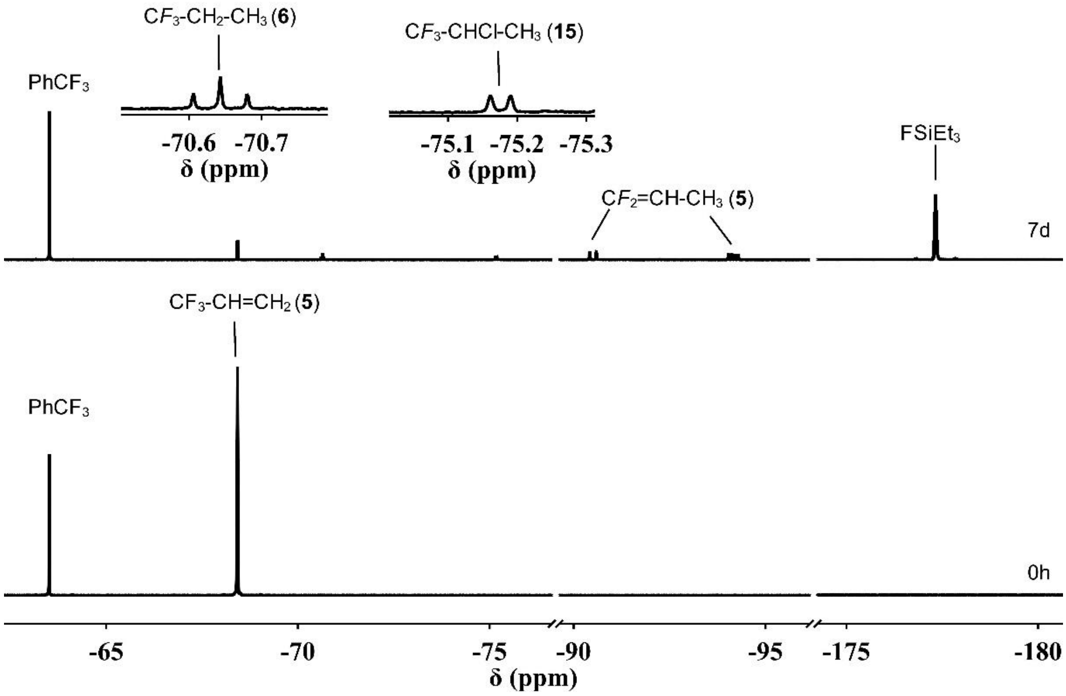

Figure 20: ^{19}F NMR spectra for the activation of **14** in the presence of ACF as the catalyst and $HSiEt_3$ as the hydrogen source in C_6D_{12}.

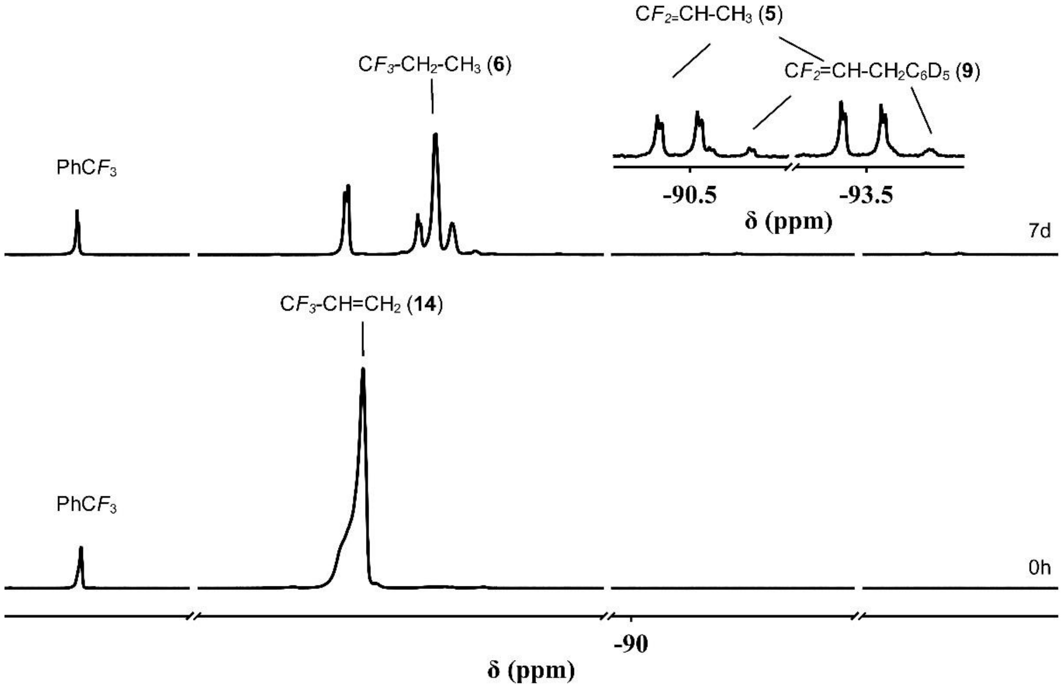

Figure 21: ^{19}F NMR spectra for the activation of **14** in the presence of ACF as the catalyst and $HSiEt_3$ as the hydrogen source in C_6D_6.

Analytical data of $CF_3CF=CH_2$ (1) [1]:

δ ^{1}H NMR (300 MHz, C_6D_{12}): 4.46 (dd, *J* = 13.2 Hz, *J* = 4.9 Hz, 1H, CH_2), 4.32 (ddq, *J* = 14.8 Hz, *J* = 4.9 Hz, *J* = 1.6 Hz, 1H, CH_2) ppm

δ ^{19}F NMR (282 MHz, C_6D_{12}): -74.4 (dd, *J* = 11 Hz, *J* = 1.6 Hz, 3F, CF_3), -125.2 (ddq, *J* = 44 Hz, *J* = 14.8, *J* = 11 Hz, 1F, CF) ppm

δ ^{19}F{^{1}H} NMR (282 MHz, C_6D_{12}): -74.4 (d, *J* = 11 Hz, 3F, CF_3), -125.2 (q, *J* = 11 Hz, 1F, CF) ppm

Analytical data of $CF_2=CFCH_3$ (2) [2]:

δ ^{1}H NMR (300 MHz, C_6D_6): 3.87 (dt, *J* = 17 Hz, *J* = 4.2 Hz, 3H, CH_3) ppm

δ ^{19}F NMR (282 MHz, C_6D_6): -107.4 (ddq, *J* = 91 Hz, *J* = 32 Hz, *J* = 4.2 Hz, 1F, CF_2), -127.2 (ddq, *J* = 114 Hz, *J* =91 Hz, *J* = 4.2 Hz, 1F, CF_2), -168.2 (ddq, *J* = 114 Hz, *J* = 32 Hz, *J* = 17 Hz, 1F, CF) ppm

δ $^{19}F\{^1H\}$ NMR (282 MHz, C_6D_6): -107.4 (dd, *J* = 91 Hz, *J* = 32 Hz, 1F, CF_2) , -127.2 (dd, *J* = 114 Hz, *J* = 91 Hz, 1F, CF_2), -168.2 (dd, *J* = 114 Hz, *J* = 32 Hz, 1F, CF) ppm

Analytical data of $CF_2CFCH_2C_6D_5$ (3):

δ ^1H NMR (300 MHz, C_6D_6): 3.10 (ddd, *J* = 23 Hz, *J* = 4 Hz, *J* = 3 Hz, 3H, CH_3) ppm

δ ^{19}F NMR (282 MHz, C_6D_6): -107 (ddt, *J* = 87 Hz, *J* = 32 Hz, *J* = 3 Hz, 1F, CF_2), -125.7 (ddt, *J* = 114.7 Hz, *J* = 86.4 Hz, *J* = 4.4 Hz, 1F, CF_2), -173 (ddt, *J* = 114.7 Hz, *J* = 32 Hz, *J* = 23 Hz, 1F, CF) ppm

δ $^{19}F\{^1H\}$ NMR (282 MHz, C_6D_6): -107 (dd, *J* = 87 Hz, *J* = 32 Hz, 1F, CF_2), -125.7 (dd, *J* = 114.7 Hz, *J* = 86.4 Hz, 1F, CF_2), -173 (ddt, *J* = 114.7 Hz, *J* = 32 Hz, *J* = 23 Hz, 1F, CF) ppm

Analytical data of $CF_3CH{=}CHF$ (4a) [3]**:**

δ ^1H NMR (300 MHz, C_6D_{12}): 6.24 (ddq, *J* = 77 Hz, *J* = 11 Hz, *J* = 2 Hz, 1H, CH), 4.88 (dqd, *J* = 15 Hz, *J* = 11 Hz, *J* = 7 Hz, 1H, CH) ppm

δ ^{19}F NMR (282 MHz, C_6D_{12}): -62.3 (tt, *J* = 8 Hz, *J* = 2 Hz, 3F, CF_3), -119.3 (dm, *J* = 77, *J* = 8 Hz, 1F, CF) ppm

δ $^{19}F\{^1H\}$ NMR (282 MHz, C_6D_{12}): -61.5 (d, *J* = 8 Hz, 3F, CF_3), -119.3 (q, *J* = 8 Hz, 1F, CF) ppm

Analytical data of $CF_3CH{=}CHF$ (4b) [3]**:**

δ ^1H NMR (300 MHz, C_6D_{12}): 5.42 (dd, *J* = 77 Hz, *J* = 5 Hz, 1H, CH), 4.26 (dqd, *J* = 37 Hz, *J* = 7.7 Hz, *J* = 5 Hz, 1H, CH) ppm

δ ^{19}F NMR (282 MHz, C_6D_{12}): -58.7 (dd, *J* = 7 Hz, *J* = 17 Hz, 3F, CF_3), -110 (dm, *J* = 77, *J* = 17 Hz, 1F, CF) ppm

δ $^{19}F\{^1H\}$ NMR (282 MHz, C_6D_{12}): -58.7 (d, *J* = 17 Hz, 3F, CF_3), -110.3 (q, *J* = 17, 1F, CF) ppm

Analytical data of CF_2CHCH_3 (5):

δ ^1H NMR (300 MHz, C_6D_6): 4.00 (dqd, *J* = 25 Hz, *J* = 7 Hz, *J* = 2 Hz, 1H, CH), 1.48 (dt, *J* = 7 Hz, *J* = 3 Hz, 3H, CH_3) ppm

δ ^{19}F NMR (282 MHz, C_6D_6): -90.4 (m, 1F, CF_2), -94.1 (ddq, *J* = 48 Hz, *J* = 25 Hz, *J* = 3 Hz, 1F, CF_2) ppm

δ $^{19}F\{^1H\}$ NMR (282 MHz, C_6D_6): -90.4 (d, *J* = 48 Hz, 1F, CF_2), -94.1 (d, *J* = 48 Hz, 1F, CF_2) ppm

Analytical data of $CF_3CH_2CH_3$ (6):

δ ^1H NMR (300 MHz, C_6D_6): 1.95 (m, 2H, CH_2), 1.06 (t, *J* = 8 Hz, 3H, CH_3) ppm

δ ^{19}F NMR (282 MHz, C_6D_6): -70.5 (t, *J* = 10 Hz, 3F, CF_3) ppm

δ $^{19}F\{^1H\}$ NMR (282 MHz, C_6D_6): -70.5 (s, 3F, CF_3) ppm

Analytical data of CF_2=$CHCH_2C_6D_5$ (9):

δ ^1H NMR (300 MHz, C_6D_6): 3.99 (dtd, *J* = 22.4 Hz, *J* = 8 Hz, *J* = 2.1 Hz, 1H, CH), 2.94 (dt, *J* = 22.4 Hz, *J* = 8 Hz, 2H, CH_2) ppm

δ ^{19}F NMR (282 MHz, C_6D_6): -90.3 (d, *J* = 46 Hz, 1F, CF_2), -93.1 - -93.7 (m, 1F, CF_2) ppm

δ $^{19}F\{^1H\}$ NMR (282 MHz, C_6D_6): -90.3 (d, *J* = 46 Hz, 1F, CF_2), -93.1 - -93.7 (d, *J* = 46 Hz, 1F, CF_2) ppm

Analytical data of CF_3CHFCH_2F (10a):

δ ^1H NMR (300 MHz, C_6D_6): 4.84 (m, 1H, CHF), 4.66 (m, 1H, CH_2F), 4.76 ppm (m, 1H, CH_2F) ppm

δ ^{19}F NMR (282 MHz, C_6D_6): -79.6 (q, *J* = 6 Hz, *J* = 11 Hz, *J* = 25 Hz, 3F, CF_3), -208.3 (m, 1F, CHF), -237.3 (m, 1F, CH_2F) ppm

δ $^{19}F\{^1H\}$ NMR (282 MHz, C_6D_6): -79.6 (q, *J* = 6 Hz, *J* = 11 Hz, *J* = 16 Hz, 3F, CF_3), -208.3 (qt, *J* = 6 Hz, *J* = 11 Hz, *J* = 16 Hz, 1F, CHF), -237.3 (m, 1F, CH_2F) ppm

Analytical data of $CF_3CF_2CH_3$ (10b):

δ ^1H NMR (300 MHz, C_6D_6): 1.70 (tt, *J* = 1.2 Hz, *J* = 18 Hz, 3H, CH_3), ppm

δ ^{19}F NMR (282 MHz, C_6D_6): -88.3 (s, 3F, CF_3), -112.8 (q, *J* = 18 Hz, 2F, CF_2) ppm

δ $^{19}F\{^1H\}$ NMR (282 MHz, C_6D_6): -88.3 (s, 3F, CF_3), -112.8 (s, *J* = 18 Hz, 2F, CF_2) ppm

Analytical data of $CF_3CH_2CHF_2$ (10c):

δ ^1H NMR (300 MHz, C_6D_6): 2.66 (m, *J* = 4.7 Hz, 3H, CH_2), 5.97 (tt, *J* = 4.7 Hz, *J* = 54 Hz, 1H, CHF_2) ppm

δ ^{19}F NMR (282 MHz, C_6D_6): -63.1 (tm, *J* = 8 Hz, 3F, CF_3), -116 (dm, *J* = 8 Hz, *J* = 57 Hz, 2F, CHF_2) ppm

δ ^{19}F{^{1}H} NMR (282 MHz, C_6D_6): -63.1 (t, *J* = 8 Hz, 3F, CF_3), -116 (m, *J* = 8 Hz, 2F, CHF_2) ppm

Analytical data of $CF_3CHFCH_2C_6D_5$ (11):

δ ^{1}H NMR (300 MHz, C_6D_6): 2.25 (d, *J* = 6 Hz, 2H, CH_2), 3.94 (t, *J* = 6 Hz, 1H, CH) ppm

δ ^{19}F NMR (282 MHz, C_6D_6): -79.4 (dd, *J* = 6 Hz, *J* = 11 Hz, 3F, CF_3), -202 (m, *J* = 18, 1F, CF) ppm

δ ^{19}F{^{1}H} NMR (282 MHz, C_6D_6): -79.4 (d, *J* = 11, 3F, CF_3), -202 (q, *J* = 11, 1F, CF) ppm

Analytical data of $CF_3CDFCH_2C_6D_5$ (12):

δ ^{1}H NMR (300 MHz, C_6D_6): 2.28 (d, *J* = 6 Hz, 2H, CH_2), ppm

δ ^{19}F NMR (282 MHz, C_6D_6): -80.7 (dd, *J* = 6 Hz, *J* = 11 Hz, 3F, CF_3), -200 (m, *J* = 18, 1F, CF) ppm

δ ^{19}F{^{1}H} NMR (282 MHz, C_6D_6): -80.7 (d, *J* = 11, 3F, CF_3), -200 (q, *J* = 11, 1F, CF) ppm

Analytical data of CF_3CHFCH_3 (13):

δ ^{1}H NMR (300 MHz, C_6D_6): 1.5 (dd, *J* = 25 Hz, *J* = 6 Hz, 3H, CH_3), 4.8 (dm, *J* = 46 Hz, *J* = 6 Hz, 1H, CH) ppm

δ ^{19}F NMR (282 MHz, C_6D_6): -83.5 (dd, *J* = 11 Hz, *J* = 6 Hz, 3F, CF_3), -195.8 (m, *J* = 10 Hz, 1F, CF) ppm

δ ^{19}F{^{1}H} NMR (282 MHz, C_6D_6): -82.3 (d, *J* = 11 Hz, 3F, CF_3), -194.5 (q, *J* = 10 Hz, 1F, CF) ppm

Analytical data of $CF_3CH{=}CH_2$ (14):

δ ^{1}H NMR (300 MHz, C_6D_6): 5.39-5.33 (m, 2H, CH_2), 4.88 - 4.75 (m, 1H, CH) ppm

δ ^{19}F NMR (282 MHz, C_6D_6): -67.4 (d, *J* = 4 Hz, 3F, CF_3) ppm

δ ^{19}F{^{1}H} NMR (282 MHz, C_6D_6): -67.4 (s, 3F, CF_3) ppm

Analytical data of $CF_3CHClCH_3$ (15) [4]:

δ ^{1}H NMR (300 MHz, C_6D_6): 4.04 (q, *J* = 6 Hz, 1H, CH), 1.37 (d, *J* = 6 Hz, 3H, CH_3) ppm

δ ^{19}F NMR (282 MHz, C_6D_6): -74.2 (d, *J* = 10 Hz, 3F, CF_3) ppm

δ ^{19}F{^{1}H} NMR (282 MHz, C_6D_6): -74.2 (s, 3F, CF_3) ppm

References:

(1) Stepanov, I. V.; Burmakov, A. I.; Kunshenko, B. V.; Alekseeva, L. A.; Yagupol'skii, L. M. *Zh. Org. Khim* **1983**, *19*, 273–279

(2) Meißner, G.; Kretschmar, K.; Braun, T.; Kemnitz, E. *Angew. Chem. Int. Ed.* **2017**, *56* (51), 16338–16341. doi:10.1002/anie.201707759

(3) Kühnel, M. F.; Lentz, D. *Angew. Chemie - Int. Ed.* **2010**, *49* (16), 2933–2936. doi:10.1002/anie.200907162

(4) Hanack, M.; Ullmann, J. *J. Org. Chem.* **1989**, *54* (6), 1432–1435. doi:10.1021/jo00267a036
